# Biomimetic nanodelivery system with simultaneous blood–brain barrier-crossing and neuroprotective abilities for anti-parkinsonian therapy

**DOI:** 10.1186/s13020-025-01239-2

**Published:** 2025-11-08

**Authors:** Xuanying Yin, Jinmei Qiu, Guowang Cheng, Jiaxin Wu, Chen Wang, Chunye Zheng, Shuiqing Huang, Tongkai Chen

**Affiliations:** 1 Science and Technology Innovation Center, Guangzhou University of Chinese Medicine, 12 Jichang Road, Guangzhou, 510405 China; 2 Department of Neurology, The Second Affiliated Hospital of Guangzhou University of Chinese Medicine, Guangzhou, 510120 China

**Keywords:** Parkinson’s disease, Neurodegeneration, Neuroinflammation, Brain targeting, Biomimetic nanomedicine

## Abstract

**Background:**

Parkinson’s disease (PD) has emerged as a critical public health challenge amidst global population aging. The pathogenesis of PD is extremely complex. Notably, evidence showed that neuroinflammation due to microglial activation is a critical driver of dopaminergic neuron loss in patients with PD. Therefore, several strategies aimed at alleviating neuroinflammation are currently being tested for the treatment of PD. However, current anti-inflammatory agents exhibit limited therapeutic efficacy in vivo due to hindrances caused by the blood–brain barrier (BBB). To overcome BBB-related challenges, we developed a biomimetic nanodelivery system (DCM@Nar-NCs) by encapsulating naringenin nanocrystals (Nar-NCs) within differentiated HL-60 cell membranes. Our analysis demonstrated that DCM@Nar-NCs could act as an innovative nanoplatform for PD therapy, showing BBB penetration capabilities and exhibiting precise accumulation at sites of neuroinflammation. This targeted delivery enables more precise and potent treatment than existing therapeutic modalities.

**Methods:**

The BBB penetration efficiency and brain-targeted delivery of DCM@Nar-NCs were assessed both in vitro and in vivo. The neuroprotective effects were comprehensively investigated in cellular and animal levels. Finally, the ability of DCM@Nar-NCs to ameliorate motor dysfunction and cognitive impairment was validated in 1-methyl-4-phenyl-1,2,3,6-tetrahydropyridine (MPTP)-induced PD mouse models.

**Results:**

DCM@Nar-NCs exhibited significantly enhanced BBB permeability and could exert dual therapeutic effects. Notably, DCM@Nar-NCs modulated microglial polarization (pro-inflammatory M1 phenotype to neuroprotective M2 phenotype), thereby attenuating neuroinflammatory cascades. Additionally, DCM@Nar-NCs could ameliorate mitochondrial dysfunction and thereby prevent the apoptosis and destruction of dopaminergic neurons. Finally, behavioral assessments in animal models confirmed the remarkable capacity of DCM@Nar-NCs to reverse PD-related motor deficits and cognitive impairment.

**Conclusion:**

Collectively, the novel PD treatment approach developed in this study offers superior biosafety and treatment efficacy, highlighting its strong potential for clinical translation.

**Graphical Abstract:**

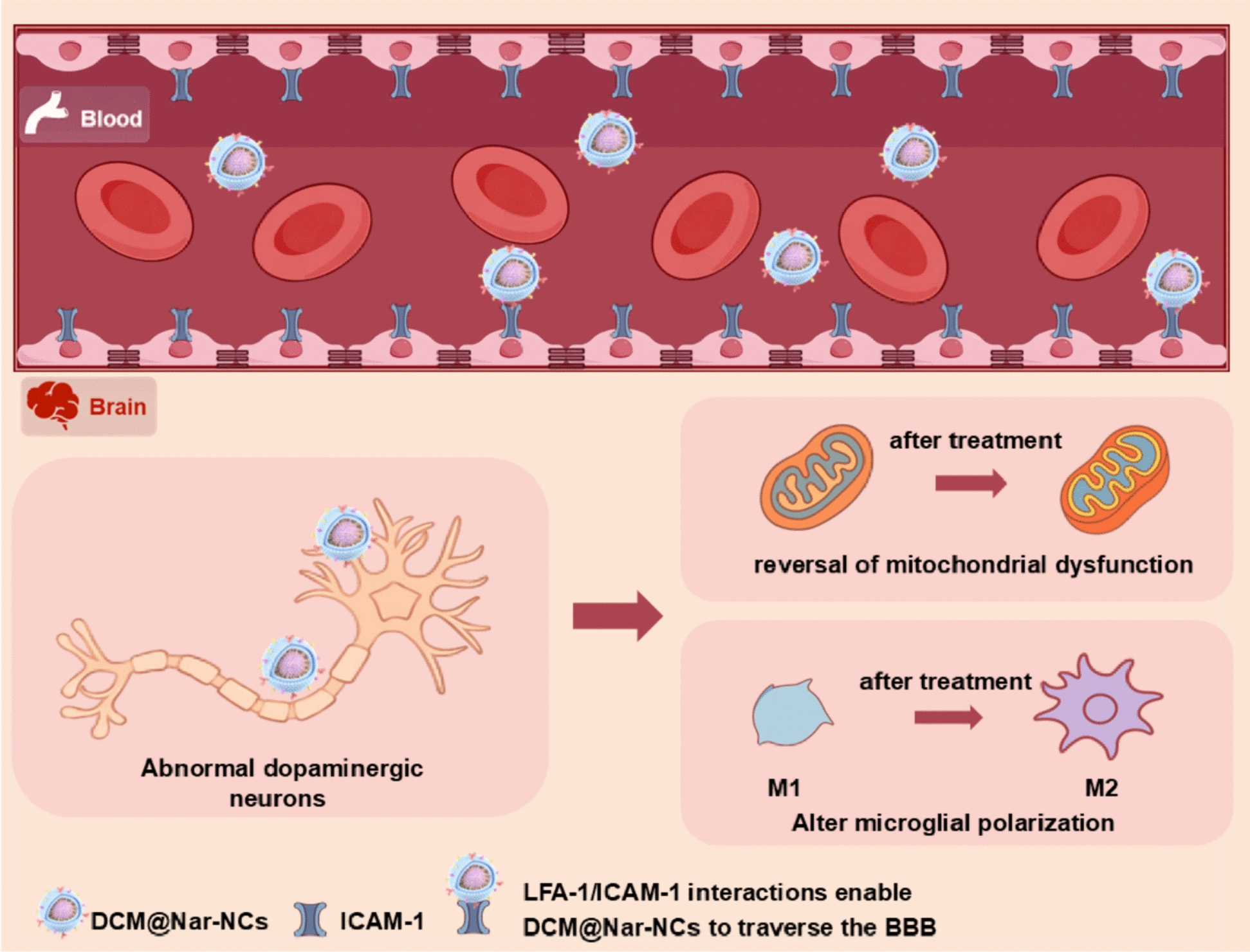

**Supplementary Information:**

The online version contains supplementary material available at 10.1186/s13020-025-01239-2.

## Introduction

Parkinson’ s disease (PD), the second most prevalent neurodegenerative disorder globally, was estimated to affect over 8.5 million people worldwide in 2019 [1]. Notably, men are approximately twice as susceptible to PD as women. Although the incidence of this condition peaks between 85 and 94 years of age, the age-standardized rates vary more than five-fold across different regions, reflecting variations in life expectancy, environmental exposures, and genetic backgrounds [2].

PD pathology involves oxidative stress, neuroinflammation, mitochondrial dysfunction, protein homeostasis disruption, endoplasmic reticulum stress, and gut dysbiosis [3]. However, the failure of mitochondrial bioenergetics, accompanied by microglial activation and cellular senescence, is regarded as the primary driver of PD [4]. Misfolded α-synuclein (α-syn) and PINK1/Parkin defects trigger Complex I failure, disrupting the membrane potential and depleting ATP in substantia nigra (SN) neurons [5]. Consequently, the elevated reactive oxygen species (ROS) promote the release of damage-associated molecular patterns (DAMPs), engaging pattern recognition receptors (PRRs) (e.g., CD36 and Fyn kinase complexes) [6]. The activation of PRRs causes microglia to shift from a homeostatic (M0) to a pro-inflammatory M1 phenotype. Toll-like receptor (TLR) ligands further sustain M1 activation, driving dopaminergic neuron loss through the secretion of tumor necrosis factor (TNF)-α, interleukin (IL)-1β, and IL-6 [7]. The activated M1 microglia prime nuclear factor (NF)-κB to assemble the NLRP3 inflammasome, activate caspase-1 to release IL-1β and IL-18, disrupt mitophagy, and promote ROS accumulation [6]. Notably, multiple factors regulate microglial polarization, including mitochondrial complex I-derived ROS [8], impaired Nrf2 signaling [6], NF-κB-mediated cytokine cascades [9], SIRT3/AMPK dysregulation [10], and lysine acetylation-dependent transport deficits [11]. In contrast, IL-4 and IL-13 drive M2 polarization through STAT6 [12], PPARγ [13], KLF4 [14], and c-Myc [15], upregulating the expression of IL-10 [16], transforming growth factor (TGF)-β [16], insulin-like growth factor (IGF)-1 [17], brain-derived neurotrophic factor (BDNF) [18], and phagocytic receptors (CD206, Arg1), thereby enhancing debris clearance and promoting tissue regeneration [16]. This energy–inflammation loop highlights that restoring mitophagy and promoting M2 polarization are essential for halting PD progression.

Current dopaminergic agents, anticholinergics, monoamine oxidase B (MAO-B) inhibitors, and catechol-O-methyltransferase (COMT) inhibitors relieve PD symptoms [19], but neither halt PD progression nor cross the blood–brain barrier (BBB) to deliver anti-inflammatory therapies. This highlights the urgent need for disease-modifying and anti-inflammatory interventions for PD management [20]. Nanotechnology enables the precise delivery of neuroprotective agents across the BBB for targeted PD therapy [21]. Numerous nanoplatforms (e.g., liposomes, polymeric nanoparticles, micelles, and metal organic framework) have been developed to enhance brain uptake and target dopaminergic neurons or microglia by leveraging mechanisms such as receptor-mediated transcytosis and adsorptive endocytosis [22]. To enhance delivery to the brain parenchyma, several nanosystem-based strategies have been implemented. These include photothermal modulation [23], focused ultrasound-mediated BBB disruption [24], intranasal delivery [25], and biomimetic approaches [26]. Concurrently, nanocrystals have gained prominence for CNS drug delivery due to their drug-loading capacity, enhanced dissolution rates, and tunable release kinetics [27]. Meanwhile, researchers are increasingly using membranes from red blood cells, white blood cells, or cancer cells to coat nanoparticles and promote self-antigen presentation, prolonged circulation, immune evasion, and targeted accumulation in inflamed tissues [28]. Therefore, encapsulating nanocrystals within cell membranes represents a valuable approach to achieve biomimetic targeting, BBB penetration, and promotes preferential accumulation at lesion sites.

**Fig. 1:**
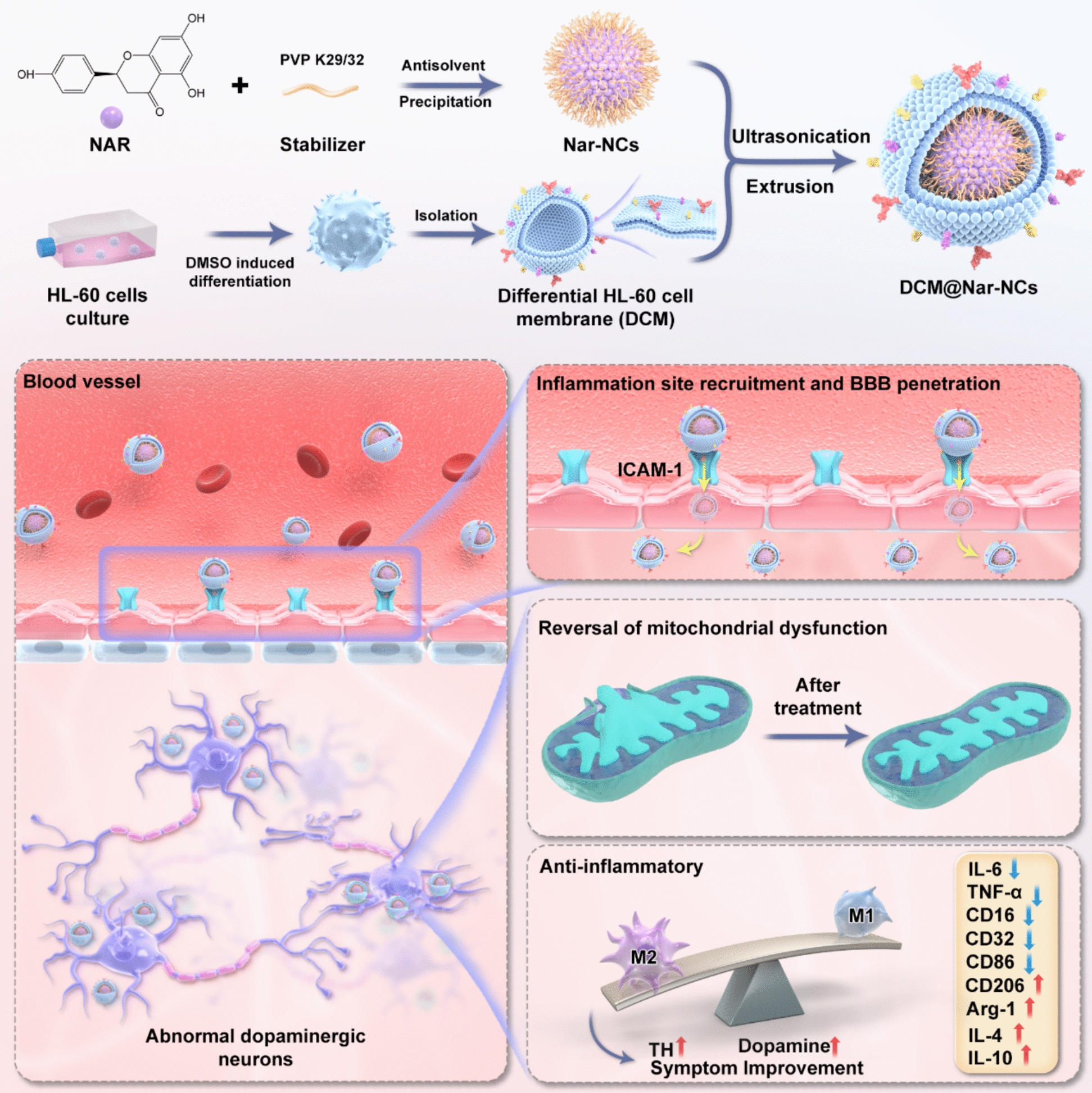
Synthesis and application of DCM@Nar-NCs for PD treatment. To prepare DCM@Nar-NCs, Nar-NCs were encapsulated within differentiated HL-60 cell membranes (DCMs). This was followed by ultrasonication and extrusion to generate the core–shell structure of DCM@Nar-NCs. Specifically, the surface of Nar-NCs was coated with DCM. The LFA-1 protein on the DCM surface could specifically bind to the ICAM-1 protein on the membranes of brain endothelial cells. This allowed the nanodelivery system to cross the BBB, target sites of inflammation, release drugs, and treat the pathological changes associated with PD. Treatment with DCM@Nar-NCs reversed mitochondrial dysfunction and ameliorated neuroinflammation, thereby improving motor function and alleviating PD-related symptoms in PD models

Naringenin (Nar), a natural flavonoid, demonstrates anti-neuroinflammatory effects by promoting M2 polarization in microglia. Moreover, it exerts neuroprotective effects by mitigating oxidative stress via the P13K/AKT pathway, validating the value of Nar in immunotherapy and chemotherapy. However, the limitation of Nar includes suboptimal pharmacokinetics, limited BBB translocation, and inadequate immunomodulatory capacity in vivo. Current nanoscale drug delivery systems fail to simultaneously achieve high drug loading capacity, targeted delivery, and microglial reprogramming [29], underscoring the necessity for an integrative delivery platform that addresses these limitations [30]. To overcome these challenges, in this study, we developed a biomimetic nanosystem (DCM@Nar-NCs) by encapsulating Nar nanocrystals (Nar-NCs) within neutrophil membranes (differentiated HL-60 cell membranes) via an ultrasound–extrusion method. The BBB-penetrating capacity of DCM@Nar-NCs and their brain-targeted delivery were confirmed using cell-based assays and animal experiments. Mechanistic studies revealed the dual therapeutic actions of DCM@Nar-NCs, mediated by (1) the suppression of pro-inflammatory cytokine release through microglial phenotype reprogramming and (2) the prevention of apoptosis in dopaminergic neurons via the attenuation of mitochondrial dysfunction (Fig. 1). Finally, behavioral assessments and neurochemical analyses demonstrated that DCM@Nar-NCs could restore striatal dopamine metabolism and attenuate motor deficits in Parkinsonian mice. Overall, this study offers a novel biomimetic nanosystem for PD treatment.

## Materials and methods

### Materials

Naringenin (Nar) was obtained from Feiyu Biotechnology Co., Ltd. (Nantong, China). Acetone was sourced from Guangzhou Chemical Reagent Factory (Guangzhou, China). A variety of polymers and surfactants—including polyvinylpyrrolidone (PVP) K29/32 and K90, Pluronic types P407, P188, and F68, polyvinyl alcohol (PVA), hydroxypropyl methylcellulose (HPMC) variants E5, E15, and E50, as well as sodium dodecyl sulfate (SDS)—were purchased from Ashland Inc. (Kentucky, USA). Sigma-Aldrich (St. Louis, USA) supplied several bioactive and analytical compounds, including 1-methyl-4-phenylpyridinium (MPP⁺), levodopa (L-DOPA), methyl-β-cyclodextrin (MβCD), chlorpromazine (CPZ), hypertonic sucrose (HS), 5-(N-ethyl-N-isopropyl)amiloride (EIPA), and a rabbit anti-tyrosine hydroxylase (TH) antibody. A range of cell tracking and analytical kits—including Lyso-Tracker, ER-Tracker, Mito-Tracker, ATP detection kits, ROS and superoxide assays, and hematoxylin and eosin (H&E) staining kits—were acquired from Aladdin Reagent Co., Ltd. (Shanghai, China). Calcein AM/PI viability kits and 2,7-dichlorodihydrofluorescein diacetate (DCFH-DA) were obtained from Beyotime Biotechnology Co., Ltd. (Beijing, China). 1-Methyl-4-phenyl-1,2,3,6-tetrahydropyridine hydrochloride (MPTP·HCl) was provided by MedChemExpress (New Jersey, USA). Dulbecco’s Modified Eagle Medium (DMEM) and fetal bovine serum (FBS) were purchased from Gibco (Grand Island, USA). The JC-1 mitochondrial membrane potential probe was sourced from Solarbio Technology Co., Ltd. (Beijing, China).

### Cell culture

Human promyelocytic leukemia (HL-60, CL0110) cells and brain microvascular endothelial (bEnd.3, CL-0598) cells were obtained from Pricella Biotechnology Co., Ltd. (Wuhan, China). Rat adrenal pheochromocytoma (PC12, FH0414) cells were purchased from Fuheng Biology Co., Ltd. (Shanghai, China). All cells were cultured in DMEM containing 10% FBS, penicillin (100 U/mL), and streptomycin (100 μg/mL). Cells were cultured in a humidified incubator at 37 °C under 5% CO₂. The culture medium was refreshed every 48 h.

### Animals and ethics approval

For in vivo assays, male C57BL/6 mice (8 weeks, 22–25 g) were procured from Beijing Vital River Laboratory Animal Technology Co., Ltd. (Beijing, China). The animals were placed in a standard SPF facility at 25 ± 2 °C under 55 ± 5% relative humidity and had free access to food and water. All animal experiments complied with The Guide for Care and Use of Laboratory Animals and received ethics approval (No. 20200505001) from the Guangzhou University of Chinese Medicine.

### Preparation of Nar-NCs and DCM@Nar-NCs

Naringenin nanocrystals (Nar-NCs) were prepared by an antisolvent precipitation method following a preliminary round of stabilizer screening [31]. The optimized procedure involved the rapid injection of 200 μL of a Nar solution (20 mg/mL in acetone) into 10 mL of aqueous PVP K29/32 (0.5 mg/mL) under continuous stirring (1000 rpm) at ambient temperature. For the fabrication of DCM@Nar-NCs, Nar-NCs were incubated with membranes extracted from differentiated HL-60 cells (DCMs). Electrostatic interactions facilitated the spontaneous adsorption of the cell membranes onto the surface of Nar-NCs. Sonication was applied to improve coating efficiency and ensure uniform membrane encapsulation.

### Characterization

The morphology of Nar-NCs and DCM@Nar-NCs was visualized using transmission electron microscopy (TEM). Particle size distribution and zeta potential measurements were conducted using dynamic light scattering (DLS) analysis. To elucidate the self-assembly behavior of Nar with PVP K29/32, molecular dynamics simulations were performed using GROMACS version 2018.4 [32]. Successful membrane coating was validated by SDS-PAGE, which confirmed the retention of membrane proteins on DCM@Nar-NCs. Drug release kinetics were evaluated by dialyzing samples in phosphate-buffered saline (PBS; pH 7.4) at 37 °C and 100 rpm. The external medium was sampled at 0, 1, 2, 4, 8, 10, 12, 24, and 48 h, and the system was then supplemented with an equal volume of fresh medium. The Nar content in each sample was detected using a UV spectrophotometer at 260 nm.

### In vitro safety analysis

PC12 cells were added to 96-well plates (1 × 10^4^ cells per well). After 24 h, cells were exposed to escalating concentrations (0, 0.34, 0.68, 1.36, 2.72, and 5.44 μg/mL) of free Nar and Nar-NCs. Following another 24 h incubation period, cell viability was determined using the Cell Counting Kit-8 (CCK-8) as directed. In brief, 10 μL of CCK-8 reagent was added to each well, and the cells were incubated at 37 °C for 2 h. Finally, the absorbance at 450 nm was detected using a microplate reader [33].

### In vitro neuroprotective effects

#### Neuroprotection assay

PC12 cells (1 × 10^4^ cells/well) were seeded into 96-well plates and preincubated for 24 h. The cells were then treated with Nar, Nar-NCs, or DCM@Nar-NCs for 2 h prior to the addition of 2 mM MPP⁺. After a 24 h of exposure to MPP⁺, cell viability was quantified using the CCK-8 assay [34].

#### Quantification of cell viability

PC12 cells were plated in 6-well plates (2 × 10^5^ cells/well). Following a 24 h of incubation, cells were treated with the various formulations (Nar, Nar-NCs, DCM@Nar-NCs) for 2 h, and then challenged with 2 mM MPP⁺ for 24 h. Cell viability was assessed via Calcein AM/PI staining, and fluorescence signals were visualized under a fluorescence microscope. Viable cells (Calcein-AM⁺, green fluorescence) and dead cells (PI⁺, red fluorescence) were quantified using ImageJ software. Cell viability was calculated as follows [35]:

$${\text{Cell viability }} = {\text{ No}}.{\text{ of live cells}}/\left( {{\text{No}}.{\text{ of live cells }} + {\text{ No}}.{\text{ of dead cells}}} \right) \, \times { 1}00\%$$

#### Mitochondrial membrane potential analysis

PC12 cells were seeded in 6-well plates (2 × 10^5^ cells/well) and preincubated for 24 h. Treatments were applied for 2 h before the addition of 2 mM MPP⁺. After 24 h, mitochondrial membrane potential was measured with JC-1 probe. Subsequently, imaging via confocal laser scanning microscopy (CLSM) was used for imaging, and cells were quantified via flow cytometry [36].

#### Endocytosis inhibition assay

To evaluate the endocytic mechanisms, DCM@Nar-NCs were fluorescently labeled with Cy5. First, DSPE-PEG-Cy5 was incorporated into the HL-60 membrane via lipid insertion, and this mixture was incubated in the dark for 30 min. Then, unincorporated dye was removed by centrifugation to yield DCM-Cy5@Nar-NCs. PC12 cells (3 × 10^5^ cells/well) were plated in 12-well plates and incubated for 24 h. These cells were then preincubated with methyl-β-cyclodextrin (MβCD), hypertonic sucrose (HS), chlorpromazine (CPZ), or 5-(N-ethyl-N-isopropyl)-amiloride (EIPA) at 37 °C for 30 min. The medium was removed, and the cell were subsequently incubated with DCM-Cy5@Nar-NCs in serum-free medium for another 2 h. Thereafter, the cells were rinsed thrice with cold PBS, fixed with 4% paraformaldehyde, and stained with DAPI. Finally, the intracellular fluorescence intensity was imaged using CLSM.

#### Intracellular trafficking and organelle colocalization

To evaluate the intracellular distribution of DCM-Cy5@Nar-NCs, their colocalization with different organelles was visualized using lysosomal (Lyso), endoplasmic reticulum (ER), and mitochondrial (Mito) trackers. PC12 cells were seeded on confocal dishes. When they reached 80% confluence, Lyso Tracker (1:10000 dilution), ER Tracker (1:1000 dilution), and Mito Tracker (40 nM) were added, and the cells were incubated at 37 °C for 1 h, 30 min, and 1 h, respectively. Subsequently, the medium was replaced with DCM-Cy5@Nar-NCs for 2 h. The cells were then fixed and stained with DAPI, and the intracellular colocalization was observed using CLSM.

#### Mitochondrial oxygen consumption

For mitochondrial oxygen consumption analysis, the oxygen consumption rate (OCR) was measured using the Seahorse XF96 Extracellular Flux Analyzer (Seahorse Bioscience, North Billerica, MA). PC12 cells were seeded in a cell culture microplate (3.0 × 10^3^ cells/well) and allowed to adhere and grow for 24 h. Various formulations (Nar, Nar-NCs, and DCM@Nar-NCs) were added for 2 h before treatment with 2 mM MPP⁺. After 24 h, a mitochondrial stress assay was performed following treatment with various mitochondrial inhibitors: oligomycin A (1 μM), carbonyl cyanide 4-(trifluoromethoxy) phenylhydrazone (FCCP, 1 μM), and a mixture of antimycin A (1 μM) and rotenone (1 μM).

#### Mitochondrial ROS (mtROS) assay

The stock solution (5 mM in DMSO) of the MitoSOX probe was diluted to a concentration of 500 nM (working solution) by adding HBSS containing Ca^2+^ and Mg^2+^. Subsequently, the cells were treated with the working solution for 30 min and quantified via flow cytometry.

### Evaluation of in vitro anti-inflammatory activity

BV2 microglial cells (2 × 10^5^ cells/well) were plated in 12-well plates and incubated for 24 h. Cells were pretreated with Nar, Nar-NCs, or DCM@Nar-NCs for 2 h, followed by 2 mM MPP⁺ exposure for 24 h. The expression of pro-inflammatory cytokines (IL-1β, IL-6, TNF-α), anti-inflammatory cytokines (IL-4, IL-10, TGF-β), and M2 markers (Arg-1, YM-1, CD206) was subsequently measured [37].

### Evaluation of in vitro BBB transport dynamics

A Transwell-based in vitro BBB model was constructed using bEnd.3 cells (2 × 10^5^ cells/well). When a transepithelial electrical resistance (TEER) of 200 Ω cm^2^ was reached, the barrier was deemed intact. Subsequently, 20 μg/mL of DCM-Cy5@Nar-NCs or free Cy5 was added to the upper chamber. The TEER was measured before and after incubation. Finally, Cy5 concentrations in the upper and lower chambers were quantified to calculate permeability using the following formula [38]:

$$Permeability\left( \% \right) \, = \, ({1 } - {C_{\rm{1}}/{C_{{0}}) \, \times \, {1}00\%}}$$

where *C*_*0*_ and *C*_*1*_ represent the Cy5 concentrations in the upper chamber before and after incubation, respectively.

### PD mice model establishment

To establish the PD model, mice were intraperitoneally injected with MPTP·HCl (25 mg/kg/day) for five consecutive days. The MPTP was dissolved in sterile 0.9% saline prior to administration [39].

### In vivo biodistribution studies

C57BL/6 mice were randomized into two groups and received either free Cy5 (4 mg/kg) or an equivalent dose of Cy5 delivered via DCM-Cy5@Nar-NCs. Fluorescence imaging was performed at multiple time points (0, 1, 3, 6, 9, 12, and 24 h post-administration). At 6 h, mice were sacrificed and perfused. Major organs, including the heart, liver, spleen, lungs, kidneys, and brain, were collected and imaged ex vivo to evaluate fluorescence distribution.

### In vivo pharmacokinetics analysis

DCM@Nar-NCs (2.4 mg/kg) or an equal dose of Nar was injected via tail vein. Plasma samples (n = 6) were collected from the tail vein at 0.25, 0.5, 1, 2, 4, 6, 8, 12, 24, and 48 h after administration. Meanwhile, brain samples (n = 6) were collected at the same time points following perfusion. After that, LC–MS/MS was used to measure the Nar concentration in all plasma and brain tissues samples [40]. Pharmacokinetic parameters were estimated using DAS (Drug and Statistics) 2.0 software. These parameters included the peak concentration of Nar in plasma/brain (C_max_), elimination half-life (T_1/2_), area under the curve of Nar in plasma/brain from time zero to t (AUC_0-t_), and mean residence time (MRT_0-t_).

### Animal grouping and drug treatment

Male C57BL/6 J mice were randomized into six groups (n = 12/group): Control, MPTP, L-DOPA, Nar, Nar-NCs, and DCM@Nar-NCs. Mice in the Control and MPTP groups received tail vein injections of physiological saline (volumes equivalent to those used for drug administration in the other groups). L-DOPA was suspended in saline and administered intraperitoneally at a dose of 25 mg/kg. For groups receiving Nar, a dosage of 2.4 mg/kg was delivered via tail vein injection. Administration was performed once every two days over a 7-day period. Throughout the treatment phase, the body weights of the mice were recorded to monitor their general health and drug tolerability.

### Behavioral tests

Behavioral assessments were conducted daily within the light phase (09:00–12:00) at exact 24 h intervals, as follows: open-field test on day 6, pole test on day 7, rotarod test on day 8, gait analysis on day 9, Morris water maze training on days 10–14 (two sessions per day, separated by 6 h intervals) with a probe trial on day 15, and novel object recognition test across days 16–18 (habituation, familiarization, and testing, respectively). Each subject was allowed to complete only one test per day at the same time and in a uniform sequence to ensure methodological rigor and reproducibility [41].

#### Open-field test

Each mouse was placed in a transparent open field apparatus (60 cm × 60 cm × 40 cm) and allowed to explore freely for 10 min. Movement was video-recorded, and parameters including total distance traveled and mean velocity were calculated using a tracking system [42].

#### Pole test

To assess bradykinesia, mice were placed atop a vertical pole (50 cm tall). The time required to turn completely downward (T-turn) and the time to descend to the base (T-total) were recorded [43].

#### Rotarod test

Analyses of motor coordination and balance were performed with a rotarod device (7 cm diameter) rotating at a speed of 20 rpm. Each mouse was placed on the rod, and the latency to the first fall, as well as the total number of falls within a 2 min trial, were recorded [44].

#### Gait analysis

Gait dynamics were assessed using the Digigait™ Imaging System following previously described protocols [43]. Mice were initially trained to walk on a motorized treadmill belt at a constant speed of 22 cm/s. Once the mice were acclimated, their continuous locomotion within the central visual field was recorded. The software analyzed paw placement, stride parameters, and additional gait metrics.

#### Morris water maze test

Mice with MPTP-induced brain lesions show hippocampus-related spatial learning impairments. Thus, the Morris Water Maze test is a canonical paradigm for evaluating non-motor cognitive deficits and the effects of neurotherapeutic interventions in PD models [45]. In this study, the Morris Water Maze test was performed in a circular pool (diameter: 160 cm, water depth: 26 cm, water temperature: 22 ± 1 °C). A submerged escape platform (2 cm beneath the water surface) was camouflaged with titanium dioxide to prevent visual detection. The pool was virtually divided into four quadrants using automated software. During the acquisition phase, mice were placed in various quadrants and allowed to find the hidden platform. When mice failed to locate this platform within 60 s, they were guided to the platform and given 10 s to remain there. This training was repeated over five consecutive days. On the 6th day, a probe trial was conducted after removing the platform, and the mice were allowed to swim freely for 60 s. The escape latencies, swim paths, and quadrant occupancy were recorded and analyzed via automated tracking software [46].

#### Novel object recognition test

Cognitive function and recognition memory were tested in an open-top white acrylic box (50 cm × 50 cm × 50 cm). On day 1, mice were habituated to the empty arena for 10 min. On day 2, two identical objects were introduced, and mice were given 5 min to explore them. On day 3, one of the familiar objects was replaced with a novel item. Mice were then reintroduced into the arena and allowed to explore for another 5 min. Their exploration behavior and time spent exploring each object were recorded to calculate the recognition index (RI), which indicated the novelty preference [47].

### Non-motor analyses

#### Tail suspension test

Depressive-like behavior in PD mice was assessed using the tail suspension test, which was conducted in a white open-top enclosure (40 cm in height). The 1.5 cm segment at the distal end of each mouse’s tail was secured with tape and attached to a crossbar. Each mouse was suspended for 6 min, and its behavior was recorded. After the first 2 min (acclimatization phase), behavioral data were collected (final 4 min phase). Periods of active struggle and immobility were quantified from the video footage [48].

#### Nest construction

To assess daily living behavior and motivational deficits, mice were housed individually with corn cob bedding for a one-week acclimation period. On the first day of the experimental phase, six square sheets of paper (6 cm × 6 cm) were introduced into each cage. Nest building activity was monitored daily, and nests were scored on a scale of 1–4 as described previously [49].

### Pathological analysis

#### Immunohistochemical and immunofluorescence staining

Post-mortem brain tissues were collected and subjected to Nissl and TUNEL staining to assess neuronal integrity and apoptotic death, following the manufacturer’s instructions [50]. In brief, the brain tissue was cut into thin tissue sections. For Nissl staining, the hydrated tissue sections were immersed in Toluidine blue dye for 30 min and then rinsed with PBS. Subsequently, the rinsed tissue sections were subjected to differentiation in ethanol, followed by dehydration and clearing with xylene. Finally, the sections were mounted in neutral resin and imaged under an optical microscope. For TUNEL staining, dehydrated brain tissue sections were first permeabilized with 1% Triton X-100 (v/v). Subsequently, the sections were incubated with a 3% H₂O₂ blocking solution for 10 min, rinsed with PBS, and then incubated with TUNEL staining solution in a humidified chamber for 1 h. Subsequently, the nuclei were labeled with DAPI. Finally, after mounting, the stained tissue sections were imaged using a fluorescence microscope.

For immunofluorescence analysis, brain sections were blocked with 10% goat serum and then incubated at 4 °C overnight with primary antibodies against Iba-1 (1:1000) and CD86 (1:1000). Sections were subsequently washed with TBST and incubated with appropriate fluorescent secondary antibodies for 2 h at room temperature. DAPI was used for nuclear counterstaining. Images were acquired using CLSM to visualize target protein expression [51].

#### *Quantification of TH*^+^*neurons*

Brains were fixed in 4% paraformaldehyde and cryoprotected using 30% sucrose. Serial coronal Sects. (20 μm thickness) were prepared and blocked with 10% goat serum. Then, sections were incubated with an anti-TH antibody (1:2000) overnight at 4 °C, followed by a 2 h of incubation with an Alexa Fluor 594-conjugated secondary antibody at room temperature. After DAPI staining, fluorescence signals were visualized under a fluorescence microscope, and TH-positive neurons were quantified using ImageJ software [23].

#### Detection of DA and its metabolites

Striatal tissues were dissected, weighed, and homogenized in 0.4 M perchloric acid (10 μL/mg tissue). The homogenates were sonicated and centrifuged at 10,000 × g and 4 °C for 10 min. Supernatants were collected for the quantification of dopamine (DA), homovanillic acid (HVA), and 3,4-dihydroxyphenylacetic acid (DOPAC) using high-performance liquid chromatography coupled with electrochemical detection (ESA, Chelmsford, MA, USA) [52].

#### Analysis of oxidative stress

Brain tissues were homogenized in RIPA buffer containing protease and phosphatase inhibitors. The homogenate was centrifuged for 10 min at 14,000 × g and 4 °C, and the supernatant was collected. The levels of ROS, SOD, MDA, and ATP in the supernatant were quantified using ELISA kits in accordance with the manufacturers’ protocols [53].

#### Detection of macrophage polarization and inflammatory factors

RT-PCR was employed to assess the gene expression levels of M1 macrophage markers (CD86, CD16, CD32), M2 markers (CD206, Arg-1), and cytokines associated with inflammation. The mRNA levels of anti-inflammatory IL-4 and IL-10, as well as pro-inflammatory IL-6 and TNF-α, were all analyzed to determine the immune response [54].

### Biocompatibility analysis

Samples of blood and major organs were harvested from treated mice. Blood samples were used to conduct biochemical and hematological analyses. Major organs were weighed to calculate relative organ weights and were subjected to H&E staining to enable pathological examination [55].

### Statistical analysis

Data were reported as the mean ± standard deviation (SD) and analyzed with GraphPad Prism 8.0 software using the Student’s t-test, one-way analysis of variance (ANOVA), or two-way ANOVA, as appropriate. *P* < 0.05 was considered significant.

## Results and discussion

### Synthesis of DCM@Nar-NCs and assessment of physicochemical properties

To develop a nanoplatform that can cross the BBB and target sites of neuroinflammation within the brain, we first prepared Nar-NCs via the anti-solvent precipitation method. Given that surfactants and process parameters both significantly affect the physicochemical properties of Nar-NCs, systematic process optimization was conducted in the present study. First, through turbidity assessments, 0.5 mg/mL PVP K29/32 was identified as the most suitable stabilizer for nanocrystal synthesis (Fig. S1A). In subsequent single-factor experiments, we optimized the preparation of physically stable, non-aggregating nanocrystals (Fig. S1B). Finally, a stirring speed of 1000 rpm and an organic/aqueous phase ratio of 1:50 were chosen as optimal parameters for anti-solvent precipitation.

**Fig. 2:**
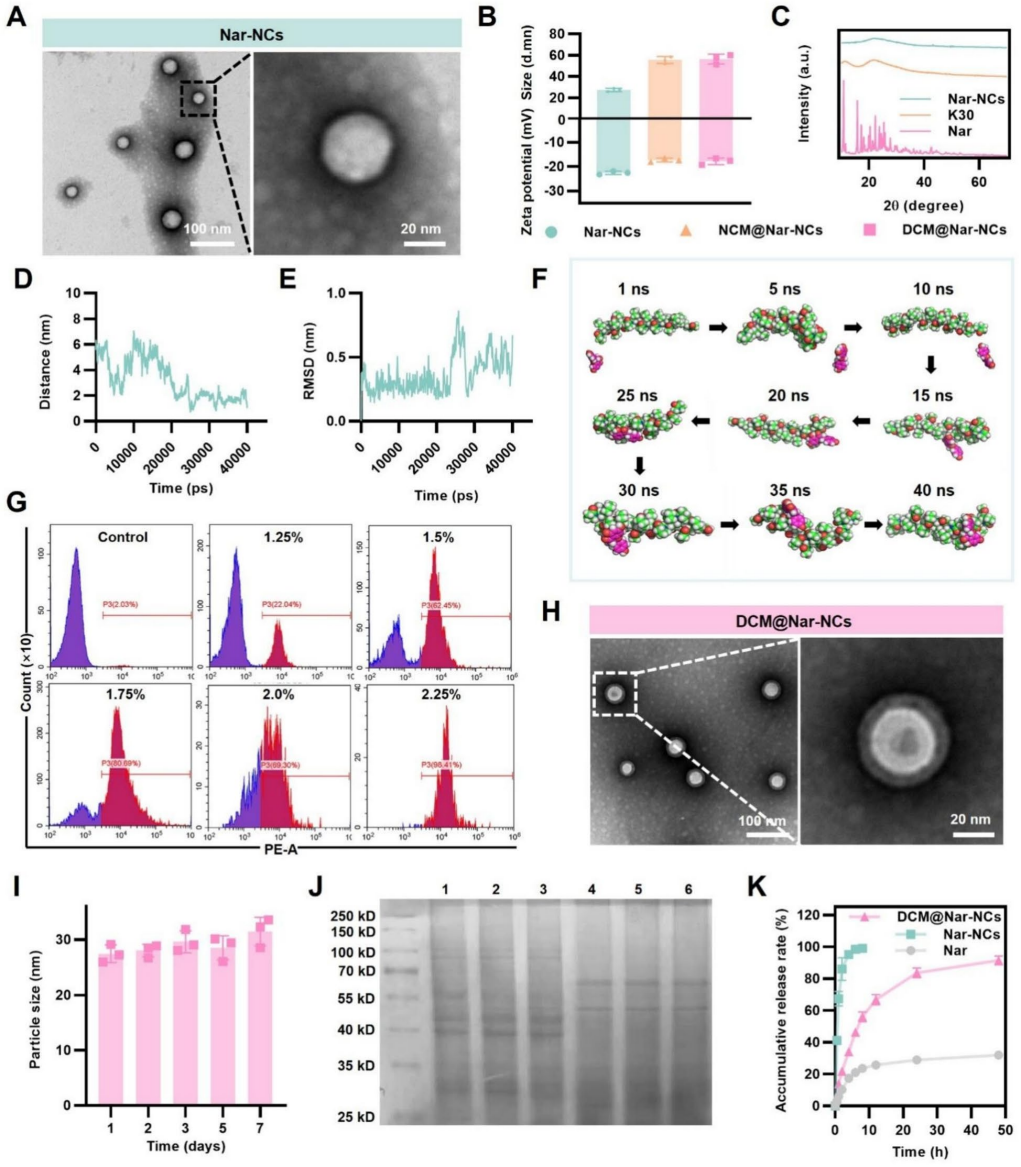
Properties of DCM@Nar-NCs. **A** TEM image of Nar-NCs. Scale bars: 100 nm and 20 nm. **B** Particle size and zeta potential of Nar-NCs, NCM@Nar-NCs, and DCM@Nar-NCs. **C** XRD spectrum of Nar-NCs. **D** Spatial distance variation of PVP K29/32 and Nar detected in the molecular dynamics simulation. **E** RMSD of the molecular system. **F** Molecular dynamics simulation of Nar and PVP K29/32 at 1, 5, 10, 15, 20, 25, 30, 35, and 40 ns. **G** Flow cytometric analysis of cell differentiation in HL-60 cells treated with different concentrations of DMSO. **H** Representative SEM image of DCM@Nar-NCs. Scale bars: 100 nm and 20 nm. **I** Particle size stability of DCM@Nar-NCs during 7 days of storage. **J** SDS-PAGE analysis of various samples: 1, DCM@Nar-NCs; 2, DCM; 3, Differentiated HL-60 cell membranes; 4, NCM@Nar-NCs; 5, NCM; and 6, Undifferentiated HL-60 cell membranes. **K** In vitro drug release profiles of Nar, Nar-NCs, and DCM@Nar-NCs

Monodisperse spherical Nar-NCs with diameters of less than 50 nm were observed using TEM (Fig. 2A). The particle size, polydispersity index (PDI), and zeta potential of Nar-NCs were found to be 27.81 ± 2.02 nm, 0.103 ± 0.014, and − 22.58 ± 0.68 mV, respectively (Fig. 2B and S2A). X-ray diffraction (XRD) analysis confirmed the transition of Nar from the crystalline state to the amorphous state following nanonization (Fig. 2C). Molecular dynamics simulation over a timescale of 40 ns showed the docking of Nar onto PVP k29/32. The center-of-mass distance decreased from 6.14 nm to 3.13 nm with 20 ns, remaining stable thereafter (Fig. 2D). Meanwhile, the root-mean-square deviation (RMSD) rose from 0.25 nm to 0.41 nm between 20 and 30 ns, reflecting conformational adjustments, and subsequently stabilized at 0.67 nm (Fig. 2E). Energy decomposition analysis revealed that van der Waals forces were the main drivers of nanocrystal assembly. These interactions were further stabilized by the formation of 1–2 persistent hydrogen bonds between the hydroxyl/carbonyl groups of Nar and the amide oxygen and nitrogen atoms of PVP K29/32 (Fig. 2F and S3).

Before coating Nar-NCs with neutrophil membranes, HL-60 cells were treated with DMSO. As a result, they differentiated into neutrophils in vitro. Using LFA-1 as a protein marker, we first examined the optimal concentration of DMSO required to induce HL-60 cell differentiation. As shown in Fig. 2G, treatment with 1.75% DMSO yielded 80.69% LFA-1-positive cells, while treatment with 2.25% DMSO increased this percentage to 98.41%. However, due to the cytotoxicity associated with higher DMSO concentrations, 1.75% was selected as the optimal concentration. Following differentiation, HL-60 cell membranes (DCMs) were extracted and used to prepare DCM@Nar-NCs via sonication and extrusion. The particle size, PDI, zeta potential, and drug loading rate of DCM@Nar-NCs were found to be 55.61 ± 3.19 nm, 0.133 ± 0.016, -17.89 ± 1.33 mV, and 92.15 ± 4.08%, respectively (Fig. 2B and S2B). After membrane coating, the particle size and zeta potential of DCM@Nar-NCs showed significant changes (Fig. S2B and 2B), confirming the successful fabrication of DCM@Nar-NCs. For control experiments, Nar-NCs were also coated with membranes derived from undifferentiated HL-60 cells (NCMs), generating NCM@Nar-NCs. TEM further demonstrated that DCMs formed a uniform coating on the surface of Nar-NCs (Fig. 2H), confirming their structural homogeneity. Moreover, the particle size of DCM@Nar-NCs remained stable over 1 week of storage, which demonstrated their good stability (Fig. 2I). Given that membrane proteins play a critical role in determining the biological fate of biomimetic nanomedicines, we assessed protein retention in DCM@Nar-NCs using the Coomassie Brilliant Blue assay. As shown in Fig. 2J, the protein band patterns of the coated biomimetic nanocrystals were similar to those of the original cell membranes. This demonstrated that DCM@Nar-NCs and NCM@Nar-NCs retained largely intact membrane proteins and thus showed the potential to penetrate the BBB and target inflammatory lesions. In drug release experiments, free Nar displayed the slowest release rate, while Nar-NCs exhibited the most rapid release (Fig. 2K). This could be attributed to the improved solubility of Nar following nanocrystal formulation. In contrast, the DCM coating introduced a physical barrier on the surface of Nar-NCs, thereby interfering with drug release. Consequently, DCM@Nar-NCs demonstrated more sustained drug release than uncoated Nar-NCs. Collectively, these findings confirmed the successful fabrication of DCM@Nar-NCs and highlighted their potential to achieve sustained drug delivery at sites of inflammatory lesions, thereby prolonging their therapeutic action.

### Ability of DCM@Nar-NCs to cross the BBB

**Fig. 3:**
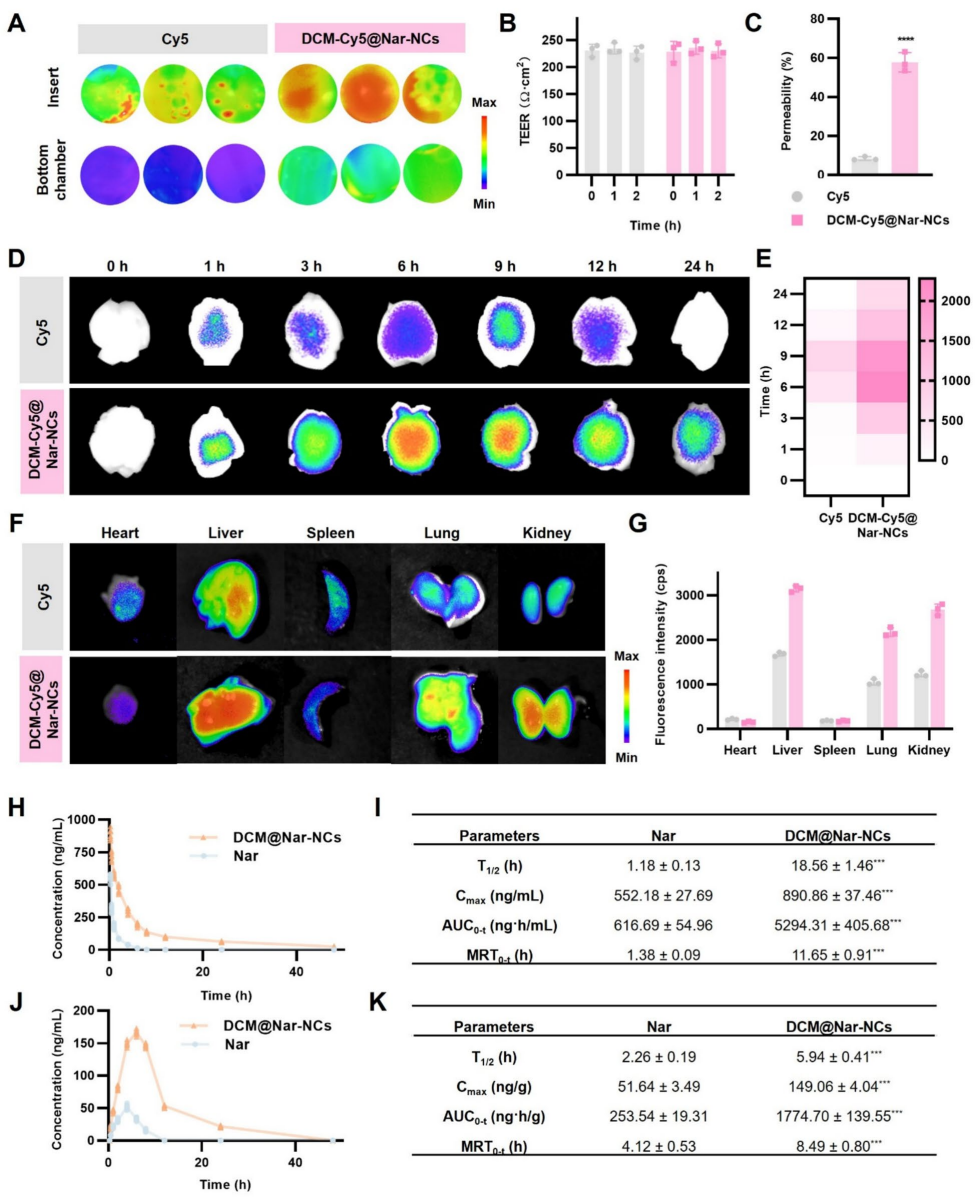
BBB-crossing capacity and biodistribution of DCM@Nar-NCs. **A** Fluorescence images of the Transwell insert and lower chamber after different treatments. **B** TEER values of bEnd.3 cell monolayers. **C** In vitro BBB-crossing ability of DCM-Cy5@Nar-NCs (n = 3). Versus the Cy5 group: ****P < 0.0001. Ex vivo fluorescence imaging of the brain (**D**) and main organs (**F**) of mice, and the corresponding quantitative analysis (**E** and **G**), respectively (n = 3). Pharmacokinetics of free Nar vs. DCM@Nar-NCs. **H** Plasma concentration–time curves following a single intravenous dose (n = 3). **H**, **J** Pharmacokinetic profiles in the plasma (**H**) and the brain (**J**) (n = 6). **I**, **K** Pharmacokinetic parameters of Nar in the plasma (**I**) and the brain (**K**) (n = 6). Versus the Nar group: ***P < 0.001

To evaluate the BBB-penetrating capacity of DCM@Nar-NCs, we first constructed an in vitro BBB model. To this end, bEnd.3 cells were seeded onto Transwell inserts [56]. Subsequently, Cy5-labeled DCM@Nar-NCs (DCM-Cy5@Nar-NCs) were introduced into the upper chambers of the inserts. After 2 h, fluorescence imaging was performed to observe the inserts and the medium in the lower chamber. As shown in Fig. 3A and C, both the inserts and the medium in the lower chamber exhibited stronger fluorescence signals in the DCM-Cy5@Nar-NCs group than in the free Cy5 group (permeability: 57.80 ± 44.95% vs. 8.47 ± 0.92%, *P* < 0.0001). Notably, the transport of DCM-Cy5@Nar-NCs did not alter the TEER of the BBB system (Fig. 3B). This indicated that the DCM-Cy5@Nar-NCs could cross the simulated BBB without compromising its integrity, thereby demonstrating high safety. Meanwhile, the increased transport across the BBB in vitro was likely due to the ICAM-1 expressed on the surface of bEnd.3 cells, which promoted the receptor-mediated transportation of this biomimetic nanosystem.

To further validate the brain-targeting capability of DCM@Nar-NCs in vivo, biodistribution studies were conducted. However, prior to this, a hemolysis test was conducted to explore the suitability of DCM@Nar-NCs for intravenous administration. As shown in Fig. S4, no significant hemolysis was observed even at high DCM@Nar-NCs concentrations (100 μg/mL), indicating that DCM@Nar-NCs could be used for intravenous injection. For the biodistribution experiments, mice received intravenous injections of DCM-Cy5@Nar-NCs and were euthanized at 0, 1, 3, 6, 9, 12, and 24 h post-injection. Then, their brain tissues were harvested for fluorescence imaging. Across all time points, the mice treated with DCM-Cy5@Nar-NCs showed significantly stronger fluorescence intensity in the brain when compared to those treated with free Cy5 (peak at 6 h: 2279.00 ± 172.10 cps vs. 492.50 ± 11.66 cps, Fig. 3D and E). Notably, by 24 h, the brain fluorescence signals were undetectable in the free Cy5 group, whereas strong signals persisted in the DCM-Cy5@Nar-NCs group (718.40 ± 35.49 cps). These findings confirmed that DCMs could enhance targeted drug delivery to brain tissues. Additionally, the evaluation of major organs at 6 h revealed that both free Cy5 and DCM-Cy5@Nar-NCs primarily accumulated in the liver, lungs, and kidneys. However, the DCM-Cy5@Nar-NCs group exhibited stronger fluorescence intensity across all organs when compared to the free Cy5 group (liver: 3148.00 ± 71.90 cps vs. 1683.00 ± 44.63 cps; lungs: 2175.00 ± 98.48 cps vs. 1053.00 ± 67.44 cps; kidneys: 2681.00 ± 126.9 cps vs. 1237.00 ± 64.26 cps; Fig. 3F and G), suggesting improved systemic circulation. Collectively, these results demonstrated that DCM@Nar-NCs, which retain functional DCM proteins, exhibit enhanced systemic circulation, brain-targeting efficiency, and BBB penetration [57]. In vivo pharmacokinetic study showed that DCM@Nar-NCs could increase the plasma T_1/2_ of Nar from 1.18 ± 0.13 h to 18.56 ± 1.46 h, Cₘₐₓ from 552.18 ± 27.69 ng/mL to 890.86 ± 37.46 ng/mL, and AUC₀–t from 616.69 ± 54.96 ng·h/mL to 5294.31 ± 405.68 ng·h/mL (*P* < 0.001; Fig. 3H, I). Meanwhile, DCM@Nar-NCs extended the brain T_1/2_ of Nar from 2.26 ± 0.19 h to 5.94 ± 0.41 h, Cₘₐₓ from 51.64 ± 3.49 ng/g to 149.06 ± 4.04 ng/g, and AUC_0–t_ from 253.54 ± 19.31 ng·h/g to 1774.70 ± 139.55 ng·h/g (*P* < 0.001; Fig. 3J, K). These results confirmed that DCM@Nar-NCs were dramatically improved systemic and brain delivery of Nar, acting as a potent platform for targeted neurotherapy.

### In vitro neuroprotective effects of DCM@Nar-NCs

A detailed understanding of cellular uptake mechanisms is crucial for understanding the biological processing of nanocarriers and the related mode of drug action. Herein, DCM-Cy5@Nar-NCs were used to explore endocytosis mechanisms and the intracellular trafficking of the nanodelivery system. The cellular uptake of DCM-Cy5@Nar-NCs by PC12 cells was observed (Fig. S5A), and the corresponding fluorescence levels were quantified (Fig. S5B). All the endocytosis inhibitors markedly reduced the cellular fluorescence intensity of DCM-Cy5@Nar-NCs, indicating that multiple endocytosis mechanisms were involved in the internalization of DCM-Cy5@Nar-NCs. Notably, macropinocytosis appeared to be predominantly responsible for the uptake of DCM-Cy5@Nar-NCs.

Subsequently, the intracellular trafficking of DCM-Cy5@Nar-NCs following internalization was examined. The colocalization of DCM-Cy5@Nar-NCs with different organelles in PC12 cells was observed (Fig. S6A), and Pearson’s correlation analysis was conducted (Fig. S6B). DCM-Cy5@Nar-NCs exhibited a large degree of fluorescence overlap with mitochondria (highest Pearson’s correlation), suggesting that these nanoparticles were mainly distributed in mitochondria following internalization.

**Fig. 4:**
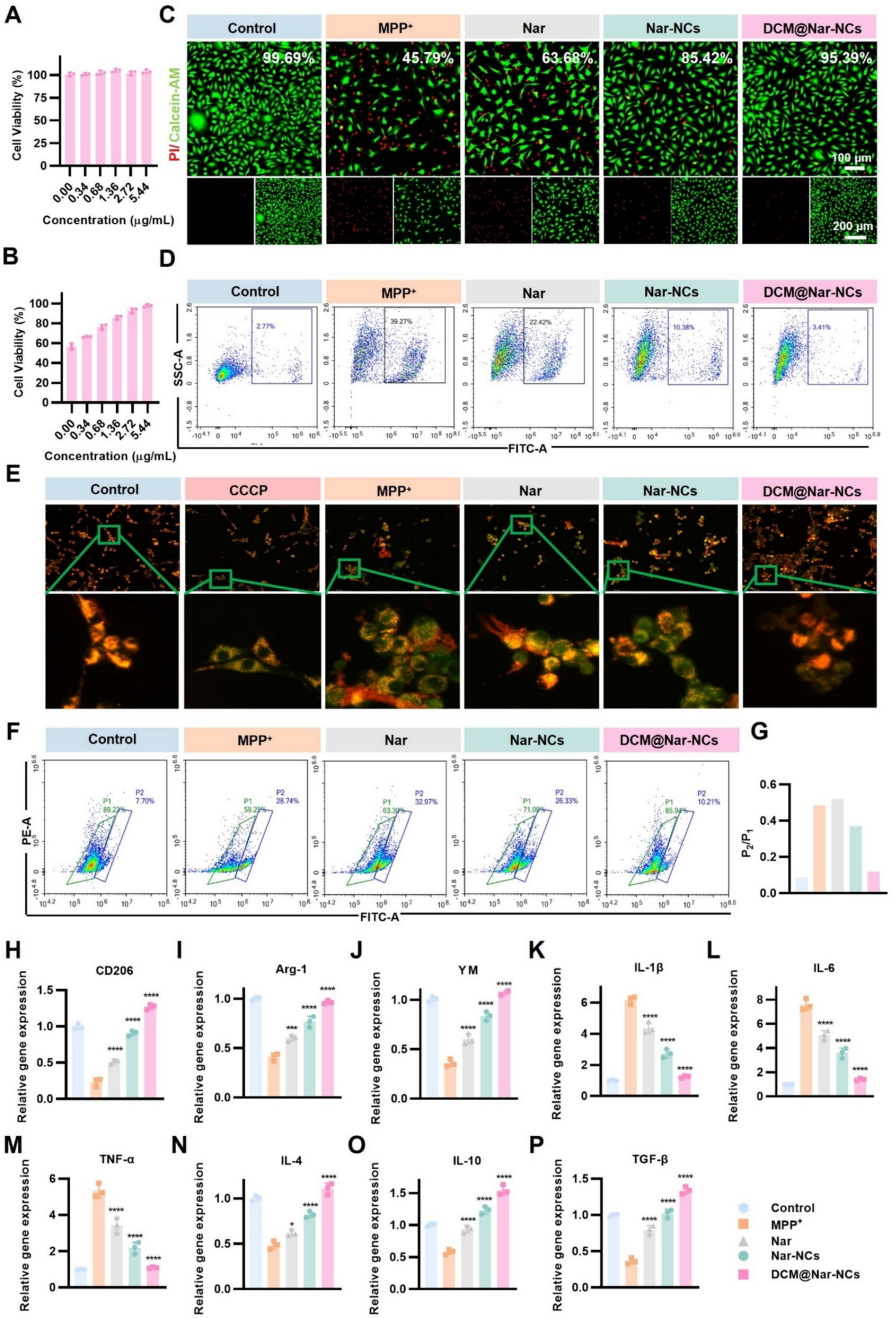
In vitro effects of DCM@Nar-NCs on neuronal apoptosis and inflammation. **A** Cytotoxicity of various concentrations of DCM@Nar-NCs in PC12 cells (n = 3). **B** Viability of MPP^+^-treated PC12 cells (n = 3). **C** Fluorescence images of live/dead PC12 cells and the quantitative analysis of cell viability (n = 3). Scale bars: 100 μm and 200 μm. **D** Flow cytometric analysis of cell apoptosis in PC12 cells. **E** Fluorescence imaging showing the mitochondrial membrane potential (MMP) of PC12 cells. **F** Flow cytometric analysis of MMP in PC12 cells after different treatments and its quantitative analysis (**G**). Levels of the M2 markers CD206 (**H**), Arg-1 (**I**), and YM (**J**) in BV2 cells (n = 3). mRNA expression of *IL-1β* (**K**), *IL-6* (**L**), *TNF-α* (**M**), *IL-4* (**N**), *IL-10* (**O**), and *TGF-β* **(P**) in BV2 cells (n = 3). Versus the MPP^+^ group: *P < 0.05, **P < 0.01, ***P < 0.001, ****P < 0.0001

.The neuroprotective and anti-neuroinflammatory effects of DCM@Nar-NCs were preliminarily evaluated in cellular models. In PC12 cells, Nar, Nar-NCs, and DCM@Nar-NCs did not exert any cytotoxic effects up to treatment concentrations of 5.44 μg/mL (Fig. 4A and S7A). The subsequent evaluation of neuroprotective effects across gradient concentrations (0.34, 0.68, 1.36, 2.72, and 5.44 μg/mL) revealed that Nar, Nar-NCs, and DCM@Nar-NCs significantly improved cell viability in cells treated with MPP⁺ (a well-established PD modeling agent). However, among the three treatment agents, Nar exhibited the weakest neuroprotective effects, likely due to its poor solubility and limited cellular uptake. In contrast, DCM@Nar-NCs demonstrated superior efficacy; this was attributed to their enhanced solubility and improved DCM-mediated cellular internalization (Fig. 4B and S7B).

These findings were validated by live/dead staining, which showed that MPP^+^ induced substantial cell death. However, DCM@Nar-NCs could effectively attenuate cellular mortality (Fig. 4C), in line with the results of the cell viability assay. To examine MPP^+^-induced apoptosis induction, Annexin V-FITC staining was conducted, and phosphatidylserine (PS) externalization was detected [58]. As shown in Fig. 4D, MPP^+^ induced significant FITC signals, whereas Nar, Nar-NCs, and DCM@Nar-NCs effectively decreased these signals. These findings revealed that Nar, Nar-NCs, and DCM@Nar-NCs could inhibit PS externalization and thus prevent apoptosis in PC12 cells. Subsequently, we examined mitochondrial dysfunction—a hallmark of ROS-induced apoptosis—using the JC-1 probe [59]. After MPP^+^ treatment, the fluorescence of mitochondria shifted from red (aggregated form, intact membrane potential) to green (monomeric form, depolarized mitochondria), with the P2/P1 ratio rising from 0.09 in control cells to 0.49 in MPP⁺‐treated cells. In contrast, DCM@Nar-NCs restored the mitochondrial membrane potential to near-normal levels, comparable to those in the control group. Specifically, the P2/P1 ratio was reduced to 0.12 after treatment with DCM@Nar-NCs (Fig. 4E–G). These results demonstrated that DCM@Nar-NCs mitigate MPP^+^-induced mitochondrial dysfunction and apoptosis in PC12 cells by scavenging excess ROS.

Given the restoration of mitochondrial membrane potential observed in the JC-1 assay, mitochondrial function was further examined to evaluate the bioenergetic status of PC12 cells following treatment. To this end, the OCR was measured (Fig. S8A). MPP⁺ exposure markedly suppressed basal respiration, ATP production, and maximal respiration in PC12 cells (Fig. S8B-8D). In contrast, treatment with Nar, Nar-NCs, and DCM@Nar-NCs effectively restored mitochondrial respiration, with DCM@Nar-NCs offering the strongest rescue effect. In parallel, mtROS levels were also assessed using the MitoSOX probe, through flow cytometry (Fig. S9A) and quantitative analysis (Fig. S9B). MPP⁺ treatment significantly increased mtROS generation in PC12 cells, whereas Nar and Nar-NCs partially reduced mtROS accumulation. However, DCM@Nar-NCs produced the most pronounced attenuation, restoring mtROS to the near-normal levels seen in the control group (Fig. S9B).

Importantly, MPP⁺ exposure markedly reduced the expression of M2 polarization markers (CD206, Arg-1, YM-1; Fig. 4H–J). For example, CD206 levels fell to 0.23 ± 0.06, Arg-1 to 0.41 ± 0.04, and YM-1 to 0.36 ± 0.04. MPP⁺ also augmented pro-inflammatory cytokine expression (Fig. 4K–M), with the levels of IL-1β, IL-6, and TNF-α rising to 6.17 ± 0.25, 7.58 ± 0.46, and 5.34 ± 0.38, respectively. Additionally, MPP⁺ attenuated anti-inflammatory mediators (Fig. 4N–P) such as IL-4, IL-10, and TGF-β, whose levels dropped to 0.49 ± 0.05, 0.58 ± 0.04, and 0.37 ± 0.04. Conversely, DCM@Nar-NCs effectively normalized these markers (Fig. 4H–P). It increased CD206, Arg-1, and YM-1 levels to 1.27 ± 0.04, 0.96 ± 0.02, and 1.08 ± 0.03; reduced IL-1β, IL-6, and TNF-α levels to 1.24 ± 0.08-, 1.42 ± 0.09, and 1.10 ± 0.04; and increased IL-4, IL-10, and TGF-β levels to 1.10 ± 0.06, 1.54 ± 0.08, and 1.34 ± 0.05. This was achieved via the abrogation of NF-κB nuclear translocation [60] and MAPK phosphorylation [61], as well as the agonistic activation of PPARγ signaling, which reactivated STAT6/PPARγ-driven M2 gene expression and anti-inflammatory cytokine secretion [62].

Meanwhile, accumulating evidence underscores the role of the gut-brain axis in PD. Peripheral inflammation and intestinal dysbiosis can amplify central neuroinflammation via circulating cytokines and vagal pathways [63]. Nar has been reported to modulate gut microbiota composition, enhance intestinal barrier integrity, and lower systemic LPS levels, thereby preventing microglial priming [64, 65]. By directly delivering Nar to lesion sites, DCM@Nar-NCs could also engage this gut–brain crosstalk to further attenuate neuroinflammation and bolster neuroprotection.

### Behavioral improvements in PD mice

The MPTP-induced murine model of PD is widely utilized in preclinical research. After crossing the BBB, MPTP is metabolized into the neurotoxin MPP^+^, which induces apoptosis in dopaminergic neurons, causes neuroinflammation, and subsequently leads to neurodegeneration, recapitulating the key pathological features of PD [66]. In this study, MPTP- induced murine models of PD were treated with Nar, Nar-NCs, and DCM@Nar-NCs to assess the therapeutic efficacy of these formulations. This was followed by comprehensive behavioral evaluations.

**Fig. 5:**
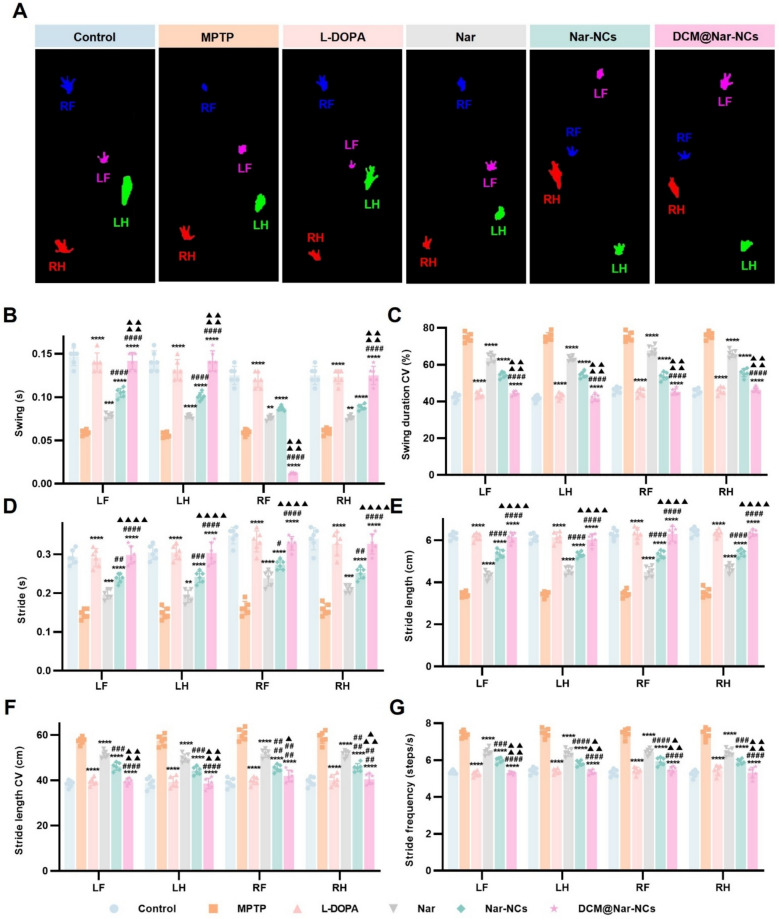
Pharmacodynamic evaluation of different treatment agents based on gait analysis. **A** Representative paw prints observed after different treatments. LF and LH: left forepaw and hindpaw, respectively; RF and RH: right forepaw and hindpaw, respectively. **B** Gait parameters such as the swing (**B**), swing duration CV (**C**), stride (**D**), stride length (**E**), stride length CV (**F**), and stride frequency (**G**) of four paws (n = 6). Versus the MPTP group: *P < 0.05, **P < 0.01, ***P < 0.001, ****P < 0.0001. Versus the Nar group: ^#^*P* < 0.05, ^##^*P* < 0.01, ^###^*P* < 0.001, ^####^*P* < 0.0001. Versus the Nar-NCs group: ^▲^*P* < 0.05, ^▲▲^*P* < 0.01, ^▲▲▲^*P* < 0.001, ^▲▲▲▲^*P* < 0.0001

Given that PD patients often experience gait abnormalities such as reduced stride length and impaired motor coordination, we employed the DigiGait Imaging System to quantify spatiotemporal gait parameters in the model mice [67]. Paw prints from the left forelimb (LF), left hindlimb (LH), right forelimb (RF), and right hindlimb (RH) were analyzed to assess gait symmetry, stability, and interlimb coordination. As shown in Fig. 5A, MPTP-exposed mice exhibited significantly shorter stride lengths and altered paw contact areas when compared to healthy controls. Therapeutic intervention, particularly with DCM@Nar-NCs, restored stride length and normalized paw contact patterns in MPTP-treated mice to levels comparable to those observed in healthy controls and mice treated with L-DOPA—the clinical gold standard for PD symptom management.

Further analysis of gait metrics—including swing phase duration, swing duration coefficient of variation (CV), stride length, stride length CV, and stride frequency — confirmed that MPTP induced marked gait instability in mice and impaired coordination. Treatment with L-DOPA, Nar, Nar-NCs, and DCM@Nar-NCs ameliorated these deficits to varying extents (Fig. 5B–G). In line with the results presented in Fig. 5A, DCM@Nar-NCs achieved comparable efficacy to L-DOPA in restoring gait performance, highlighting their potential as a promising therapeutic alternative for PD management. These findings collectively demonstrated that DCM@Nar-NCs could significantly alleviate MPTP-induced gait dysfunction in vivo.

**Fig. 6:**
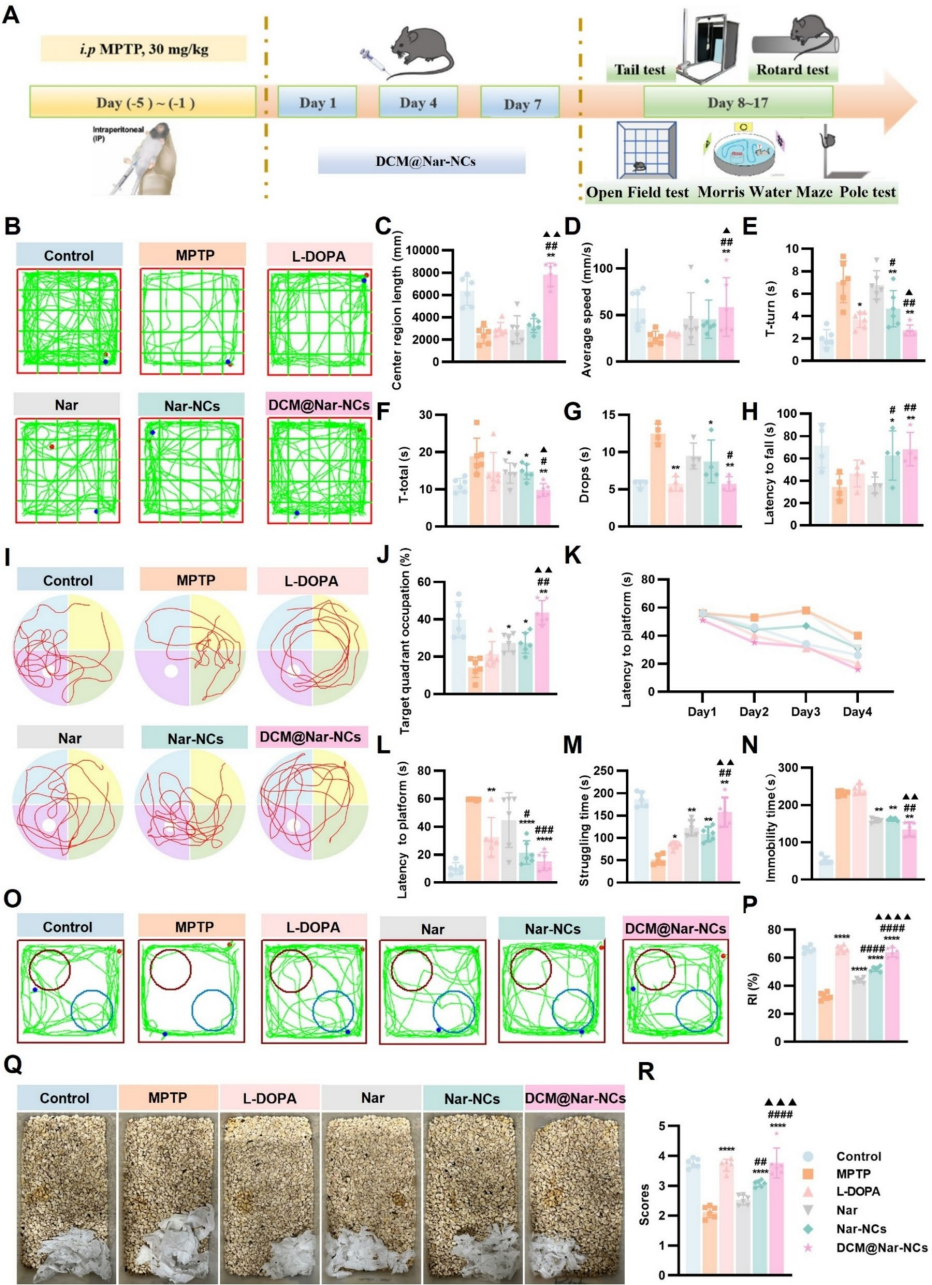
Effectiveness of DCM@Nar-NCs in alleviating MPTP-induced PD symptoms in mice. **A** Schematic diagram showing the pharmacodynamic study design. **B** Representative movement trajectories of mice during the open-field test. The distance traveled in the center (**C**) and average speed (**D**) in the open-field test (n = 6). T-turn (**E**) and T-total (**F**) values in the pole test (n = 6). Drops (**G**) and latency to fall (**H**) in the rotarod test (n = 4). **I** Representative trajectory of mice in the Morris Water Maze. **J** Target quadrant occupation in the Morris Water Maze test (n = 6). Latency to escape in each group of mice during the first four days (**K**) and on the last day (**L**) (n = 6). Struggling time (**M**) and immobility time (**N**) in the tail suspension test (n = 5). **O** Representative movement trajectory and recognition index (RI) (**P**) of mice during the novel object recognition test (n = 6). Representative images (**Q**) and scores (**R**) for nest construction in mice (n = 6). Versus the MPTP group: *P < 0.05, **P < 0.01, ***P < 0.001, ****P < 0.0001. Versus the Nar group: ^#^*P* < 0.05, ^##^*P* < 0.01, ^###^*P* < 0.001, ^####^*P* < 0.0001. Versus the Nar-NCs group: ^▲^*P* < 0.05, ^▲▲^*P* < 0.01, ^▲▲▲^*P* < 0.001, ^▲▲▲▲^*P* < 0.0001

To evaluate the therapeutic effect of DCM@Nar-NCs on other types of motor dysfunction in MPTP mice, bradykinesia [68], motor coordination and balance [69], and spontaneous movement [70] were assessed using the pole, rotarod, and open-field tests. The experimental setup for these tests is provided in Fig. 6A. In the open-field test, MPTP administration significantly reduced center-zone mobility and average speed, both of which were restored by DCM@Nar-NCs (2419.00 ± 743.60 mm vs. 7816.00 ± 1049.00 mm; 25.83 ± 6.85 mm/s vs. 58.71 ± 31.29 mm/s, *P* < 0.01) (Fig. 6B–D). In the pole test, mice treated with DCM@Nar-NCs exhibited markedly shorter total descent and turn times when compared with MPTP group. Specifically, the time to turn (T-turn) (7.06 ± 1.86 vs. 2.72 ± 0.49 s, *P* < 0.01) and the time to reach the bottom (T-total) (18.89 ± 4.87 vs. 9.83 ± 1.74 s, *P* < 0.01) both showed a decrease, indicating the reversal of bradykinesia (Fig. 6E and F). Finally, in the rotarod test, DCM@Nar-NCs reversed the dysfunction caused by MPTP, reducing the drop frequency (12.50 ± 1.29 vs. 5.75 ± 0.96, *P* < 0.01) (Fig. 6G) while increasing the fall latency (34.33 ± 11.65 vs. 68.33 ± 14.98 s, *P* < 0.01) (Fig. 6H). Thus, the mice treated with DCM@Nar-NCs demonstrated enhanced motor coordination and balance.

Cognitive deficits represent a prominent non-motor feature of PD, and current dopaminergic therapies confer negligible benefit in this domain [71]. To assess the efficacy of DCM@Nar-NCs, PD model mice were subjected to the Morris Water Maze, tail suspension, novel object recognition, and nesting tests. In the Morris Water Maze test, DCM@Nar-NCs restored target-quadrant occupancy (14.28 ± 5.33% vs. 43.69 ± 6.48%, *P* < 0.01) and reduced escape latency (59.50 ± 0.44 vs. 15.21 ± 6.56 s,* P* < 0.0001), which reflected their ability to improve spatial learning in PD mice (Fig. 6I–L). In the tail suspension test, the DCM@Nar-NCs group showed markedly increased struggling time (51.77 ± 11.08 s vs. 157.9 ± 32.68 s, *P* < 0.01) and reduced immobility time (232.40 ± 6.93 s vs. 135.00 ± 18.77 s, *P* < 0.01) than the MPTP-treated group, which reflected the alleviation of depression-like behavior (Fig. 6M,N). In the novel object recognition test, mice treated with DCM@Nar-NCs exhibited a higher discrimination index (32.39 ± 2.44 vs. 63.80 ± 3.37, *P* < 0.0001; Fig. 6O–P). The nesting test yielded similar results, and the nest-building ability of the DCM@Nar-NCs group was much stronger than that of the MPTP group (2.12 ± 0.19 vs. 3.76 ± 0.50, *P* < 0.0001; Fig. 6Q–R). These findings collectively demonstrated that DCM@Nar-NCs effectively ameliorate cognitive and affective impairments in PD models.

.Overall, our behavioral experiments comprehensively demonstrated the therapeutic effects of DCM@Nar-NCs against motor and cognitive deficits in PD mice. While both Nar and Nar-NCs conferred some measurable benefits, Nar-NCs showed better efficacy than Nar, likely due to the higher bioavailability of these nanocrystals. Nevertheless, DCM@Nar-NCs exhibited even higher efficacy than Nar-NCs, underscoring the advantages of DCM-mediated targeting.

### Histopathological assessments

**Fig. 7:**
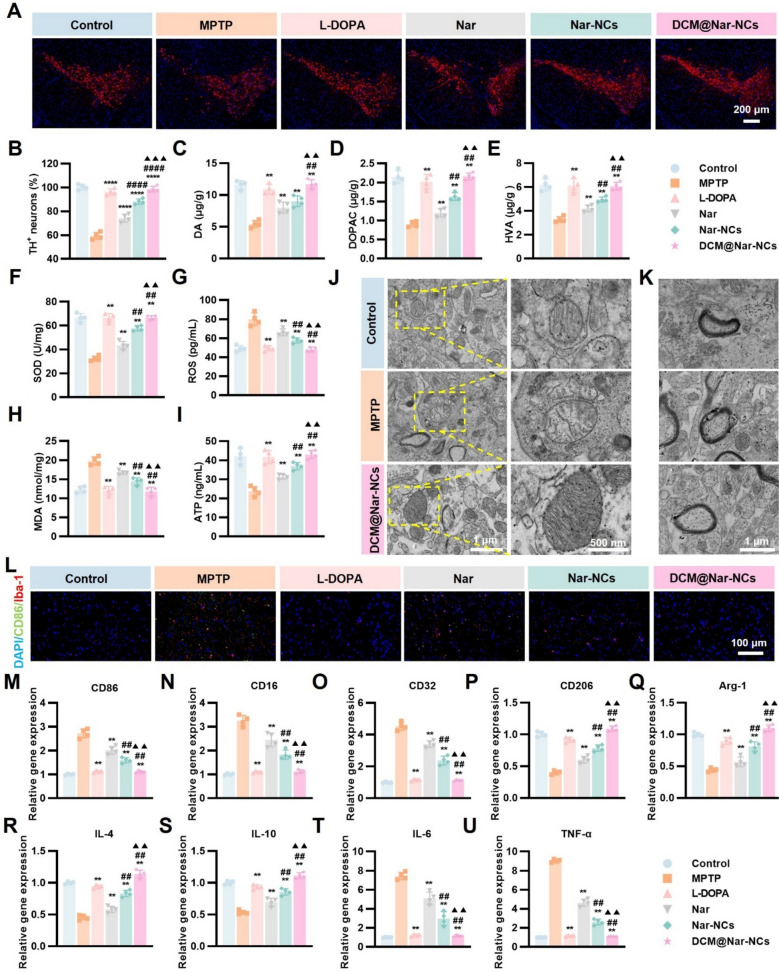
Pathological alterations in MPTP-induced PD mice subjected to DCM@Nar-NCs treatment. **A** Representative images of TH^+^ immunofluorescence staining. Scale bar: 200 μm. **B** Quantitative analysis of TH^+^ immunofluorescence staining (n = 4). Dopamine metabolism in the striatum after different treatments: DA levels (**C**), DOPAC levels (**D**), and HVA levels (**E**) (n = 4). SOD (**F**), ROS (**G**), MDA (**H**), and ATP (**I**) levels in the midbrain (n = 4). **J** Bio-TEM images of midbrain mitochondria. Scale bars: 1 µm and 500 nm. **K** Bio-TEM images of midbrain myelin sheaths. Scale bar: 1 µm. **L** Cortical expression of CD86 and Iba-1. Scale bar: 100 μm. Expression of the M1 markers CD86 (**M**), CD16 (**N**), and CD32 (**O**) and the M2 markers CD206 (**P**) and Arg-1 (**Q**) in mice subjected to different treatments (n = 4). Expression Drug release kinetics of the anti-inflammatory factors IL-4 (**R**) and IL-10 (**S**) and the pro-inflammatory factors IL-6 (**T**) and TNF-α (**U**) in mice (n = 4). Versus the MPTP group: *P < 0.05, **P < 0.01, ***P < 0.001, ****P < 0.0001. Versus the Nar group: ^#^*P* < 0.05, ^##^*P* < 0.01. Versus the Nar-NCs group: ^▲^*P* < 0.05, ^▲▲^*P* < 0.01, ^▲▲▲^*P* < 0.001

To explore the mechanism by which DCM@Nar-NCs reduce motor and cognitive dysfunction in MPTP mice, we explored the pathological microenvironment of the brain in Parkinsonian mice. Since PD is associated with the destruction of nigral dopaminergic neurons, we detected the number of dopaminergic neurons in the midbrain through immunofluorescence analysis (Fig. 7A and B). In the MPTP group, the red fluorescence (representing dopaminergic neurons) was found to be significantly weaker than that in the other groups. Meanwhile, the DCM@Nar-NCs group exhibited distinct hooked-shaped red fluorescence, indicating that DCM@Nar-NCs treatment successfully rescued nigral dopaminergic neurons.

The number of dopaminergic neurons is known to directly impact striatal levels of dopamine and its metabolites [72]. Therefore, the levels of dopamine and dopamine metabolites were examined across all groups of mice. These levels were found to be the lowest in the MPTP group. However, dopamine metabolite levels were comparable between the DCM@Nar-NCs and control groups (Fig. 7C–E), further explaining why the DCM@Nar-NCs group exhibited better motor function.

To further explore the protective effects of DCM@Nar-NCs on dopaminergic neurons in the substantia nigra on a mechanistic level, inspired by the in vitro experiments, we detected ROS levels and mitochondrial function in midbrain tissues. As shown in Fig. 7F, the levels of superoxide dismutase (SOD) were found to vary among the different treatment groups. Specifically, SOD levels were the lowest in the MPTP group (32.36 ± 2.10 U/mg). However, these levels were comparable between the DCM@Nar-NCs (66.45 ± 1.84 U/mg) and control groups (66.58 ± 3.51 U/mg). Additionally, ROS and malondialdehyde (MDA)—a product of ROS-induced lipid peroxidation [73]—showed significantly elevated levels in the MPTP group (ROS: 79.75 ± 6.63 pg/mL; MDA: 19.80 ± 1.07 nmol/mg). However, these levels were normalized in the DCM@Nar-NCs group (ROS: 48.13 ± 2.43 pg/mL; MDA: 11.75 ± 1.15 nmol/mg) (Fig. 7G and H), indicating that DCM@Nar-NCs could significantly attenuate ROS levels in the midbrain.

Additionally, we detected metabolic activity based on ATP levels and found that MPTP treatment significantly reduced mitochondrial metabolic activity in the midbrain, while DCM@Nar-NCs restored these ATP levels to normal (Fig. 7I). This suggested that DCM@Nar-NCs may attenuate the mitochondrial dysfunction caused by MPTP. To test this hypothesis, we performed ultrastructural imaging of midbrain mitochondria using bio-TEM (Fig. 7J). The control group and DCM@Nar-NCs group both exhibited clear mitochondrial membranes with internal cristae. However, the mitochondrial membranes in the MPTP group were damaged, and the cristae were significantly damaged. Moreover, MPTP destroyed the myelin sheaths of midbrain neurons. Nevertheless, this neuronal damage was also alleviated after DCM@Nar-NCs intervention (Fig. 7K). These findings showed that DCM@Nar-NCs could reverse the ROS accumulation caused by MPTP, thereby lowering oxidative stress in the brain microenvironment and ultimately protecting dopaminergic neurons.

Given the cognitive improvements observed in DCM@Nar-NCs-treated mice, we further examined the degree of neuronal apoptosis in the cerebral cortex in different groups of mice. Through Nissl and TUNEL staining, we found that DCM@Nar-NCs could reduce MPTP-induced neuronal apoptosis in the cerebral cortex (Fig. S10). Considering the role of neuroinflammation in MPTP-induced neuronal loss, we performed immunofluorescence staining for Iba-1 and CD86. Notably, the cortical expression of Iba-1 and CD86 was found to be significantly increased in the MPTP group, but it was attenuated in the DCM@Nar-NCs group (Fig. 7L). This indicated that MPTP promoted the polarization of cortical microglia from the resting state to the pro-inflammatory M1 state, likely because neuronal apoptosis in other brain regions induced the activation of DAMPs [74]. However, DCM@Nar-NCs could alleviate the excessive activation of the M1 phenotype. Furthermore, PCR analysis demonstrated that the brain tissues of MPTP mice had a more pro-inflammatory phenotype, characterized by the upregulation of CD86 (2.72 ± 0.19 vs. 1.00 ± 0.03), CD16 (3.25 ± 0.23 vs. 1.00 ± 0.03), and CD32 (4.53 ± 0.26 vs. 1.00 ± 0.03) (Fig. 7M–O) and a significant increase in the pro-inflammatory cytokines IL-6 (7.43 ± 0.38 vs. 1.00 ± 0.04) and TNF-α (9.04 ± 0.15 vs. 1.00 ± 0.02) (Fig. 7T and U). In contrast, DCM@Nar-NCs reduced these M1 markers to near-baseline levels (CD86: 1.11 ± 0.04; CD16: 1.12 ± 0.08; CD32: 1.13 ± 0.05) and lowered IL-6 and TNF-α levels to 1.15 ± 0.08 and 1.10 ± 0.04. Concomitantly, the M2 markers showed complementary changes: CD206, which fell to 0.40 ± 0.03 in MPTP mice (vs. 1.01 ± 0.04 in control), was restored to 1.09 ± 0.04 after DCM@Nar-NCs treatment. Meanwhile, Arg-1—reduced to 0.44 ± 0.03 in MPTP mice (vs. 1.00 ± 0.03 in control)—rebounded to 1.10 ± 0.05 following DCM@Nar-NCs administration (Fig. 7P, Q). The anti-inflammatory cytokines IL-4 and IL-10 rose from 0.455 ± 0.037 and 0.54 ± 0.03 to 1.14 ± 0.06 and 1.11 ± 0.04, respectively (Fig. 7R, S), while pro-inflammatory cytokines were downregulated. The serum levels of these cytokines also showed a similar trend (Fig. S11). Therefore, the results collectively indicated that DCM@Nar-NCs could reduce neuronal loss in the cortex by alleviating neuroinflammation, thereby reducing cognitive impairments in mouse models of PD.

Although this study did not include mechanistic experiments in vivo, the evidence from the literature suggests that Nar and DCM@Nar-NCs could exert neuroprotective effects by activating the following signaling pathways. First, Nar could activate the Nrf2-ARE pathway and regulate HO-1 and NQO1 to clear ROS and restore mitochondrial function [62, 75, 76]. Second, Nar could inhibit NF-κB nuclear translocation and downregulate TNF-α and IL-1β to suppress M1 microglial polarization [62, 77]. Third, Nar could promote SIRT3 expression and AMPK phosphorylation, increasing mitochondrial biogenesis and mitophagy to prevent neuronal apoptosis [78]. By delivering Nar to lesion sites in PD, DCM@Nar-NCs could likely preserve mitochondrial function and drive M2 microglial polarization via these axes. Future studies should validate the activation of these pathways in vivo via western blotting and immunofluorescence for Nrf2, NF-κB, and activated SIRT3/AMPK.

### Biocompatibility

Our aforementioned experiments indicated that DCM@Nar-NCs could significantly improve neurological function in mice with MPTP-induced PD. Nevertheless, beyond efficacy, clinical agents also need to demonstrate high levels of safety. Therefore, we evaluated the effects of DCM@Nar-NCs on blood biochemical indicators and major organs in mice. On blood routine analysis, indicators such as the white blood cell count (WBC), red blood cell count (RBC), mean corpuscular hemoglobin content (MCH), mean corpuscular hemoglobin concentration (MCHC), mean corpuscular volume (MCV), hematocrit (HCT), hemoglobin concentration (HGB), and platelet count (PLT) were found to be comparable among all groups of mice (Fig. S12). Furthermore, biochemical analysis indicated that liver and kidney function indices, including alanine aminotransferase (ALT), aspartate aminotransferase (AST), total protein (TP), albumin (ALB), blood urea nitrogen (BUN), and creatinine (CREA), did not show significant changes in any group (Fig. S13). Subsequently, upon analyzing histological changes in major organs through H&E staining, we detected no abnormal pathological changes caused by DCM@Nar-NCs treatment (Fig. S14), further demonstrating the lack of organ damage. Finally, the analysis of relative organ weights revealed no significant differences in the relative weights of the heart, liver, spleen, lungs, and kidneys among the different groups of mice (Fig. S15). These results demonstrated the excellent biocompatibility of DCM@Nar-NCs.

## Conclusions

Immune dysregulation and chronic neuroinflammation are central to PD pathogenesis. Misfolded α-syn released from dying cells binds to microglial and astrocytic PRRs, triggering the NF-κB–mediated secretion of TNF-α, IL-1β, and ROS, which exacerbate dopaminergic neuron loss. To overcome the limitations of existing therapies, in this study, we engineered a novel biomimetic nanodelivery system for PD treatment using membranes derived from HL-60 cells. We found that DCM@Nar-NCs efficiently penetrate the BBB and preferentially accumulate at neuroinflammatory loci via ICAM-1-LFA-1 mediated transcytosis, thereby enabling the targeted suppression of pro-inflammatory M1 microglia and protection of dopaminergic neurons. Compared to conventional delivery platforms, DCM@Nar-NCs demonstrated superior BBB-crossing abilities and enabled targeted modulation of neuroinflammation as well as the concomitant amelioration of both motor and cognitive dysfunction in PD models. Despite these strengths, our study has some limitations that warrant attention. First, while we demonstrated that DCM@Nar-NCs showed enhanced BBB penetration, the precise endocytic/transcytosis pathways responsible for their uptake remain unclear. Second, the system developed in this study did not specifically target dopaminergic neurons, and its effects were not evaluated in other neuronal subpopulations. Our findings relied mainly on the MPTP model and needed to be corroborated using other paradigms, such as the α-syn PFF, SNCA transgenic, 6-OHDA, and rotenone models. Third, the interplay among peripheral inflammation, gut health, and neuroinflammation via the gut–brain axis and epigenetic modulations (HDACs, miRNAs) was not explored in this study. Fourth, direct comparisons with free Nar, established anti-inflammatory agents, and other nanocarriers are needed to establish efficacy benchmarks for DCM@Nar-NCs. Finally, scalable manufacturing with validated batch reproducibility and long-term safety is still required for the clinical translation of DCM@Nar-NCs.

## Supplementary Information

Supplementary Material 1.

## Data Availability

The authors confirm that the data supporting the findings of this study are available within the article and its supplementary materials.

## Abbreviations

6-OHDA: 6-Hydroxydopamine
ALB: Albumin
ALT: Alanine aminotransferase
AMPK: AMP-activated protein kinase
ANOVA: Analysis of variance
Arg-1: Arginase-1
ARE: Antioxidant response element
AST: Aspartate aminotransferase
ATP: Adenosine triphosphate
AUC_0–t_: Area under the concentration–time curve from time zero to t
BBB: Blood–brain barrier
BDNF: Brain-derived neurotrophic factor
bEnd.3: Brain microvascular endothelial cell line
Bio-TEM: Biological transmission electron microscopy
BUN: Blood urea nitrogen
CCK-8: Cell Counting Kit-8
CD: Cluster of differentiation
CPZ: Chlorpromazine
CREA: Creatinine
CV: Coefficient of variation
Cy5: Cyanine-5
DA: Dopamine
DAMPs: Damage-associated molecular patterns
DAPI: 4’,6-Diamidino-2-phenylindole
DAS: Drug and Statistics (software)
DCFH-DA: 2’,7’-Dichlorodihydrofluorescein diacetate
DCM: Differentiated HL-60 cell membrane
DCM@Nar-NCs: DCM-coated naringenin nanocrystals
DLS: Dynamic light scatteringl
DMSO: Dimethyl sulfoxide
DOPAC: 3,4-Dihydroxyphenylacetic acid
DSPE-PEG: 1,2-Distearoyl-sn-glycero-3-phosphoethanolaminepoly( ethylene glycol)
ELISA: Enzyme-linked immunosorbent assay
EIPA: 5-(N-Ethyl-N-isopropyl)amiloride
ER-Tracker: Endoplasmic reticulum tracker
ESA: Electrochemical detection system (brand)
FBS: Fetal bovine serum
FCCP: Carbonyl cyanide-4-(trifluoromethoxy)phenylhydrazone
H&E: Hematoxylin and eosin
HBSS: Hank’s balanced salt solution
HCT: Hematocrit
HGB: Hemoglobin
HL-60: Human promyelocytic leukemia cell line
HO-1: Heme oxygenase-1
HPLC: High-performance liquid chromatography
HS: Hypertonic sucrose
HVA: Homovanillic acid
Iba-1: Ionized calcium-binding adapter molecule 1
ICAM-1: Intercellular adhesion molecule-1
IL: Interleukin
JC-1: 5,5’,6,6’-Tetrachloro-1,1’,3,3’-tetraethyl-benzimidazolylcarbocyanine iodide
KLF4: Krüppel-like factor 4
LC-MS/MS: Liquid chromatography–tandem mass spectrometry
L-DOPA: Levodopa
LFA-1: Lymphocyte function–associated antigen-1
MAPK: Mitogen-activated protein kinase
MCHC: Mean corpuscular hemoglobin concentration
MCV: Mean corpuscular volume
MDA: Malondialdehyde
miRNA: MicroRNA
Mito-Tracker: Mitochondria tracker
MPP^+^: 1-Methyl-4-phenylpyridinium
MPTP: 1-Methyl-4-phenyl-1,2,3,6-tetrahydropyridine
MRT_0–t_: Mean residence time from time zero to t
mtROS: Mitochondrial reactive oxygen species
NCM: Non-differentiated HL-60 cell membrane
NCM@Nar-NCs: NCM-coated naringenin nanocrystals
NF-κB: Nuclear factor kappa-B
NLRP3: NOD-like receptor protein 3
NQO1: NADPH quinone dehydrogenase 1
Nrf2: Nuclear factor erythroid 2–related factor 2
OCR: Oxygen consumption rate
PBS: Phosphate-buffered saline
PDI: Polydispersity index
PD: Parkinson’s disease
PI: Propidium iodide
PI3K/Akt: Phosphoinositide 3-kinase/protein kinase B
PINK1: PTEN-induced kinase 1
PLT: Platelet count
PPARγ: Peroxisome proliferator-activated receptor gamma
PRRs: Pattern recognition receptors
PVA: Polyvinyl alcohol
PVP: Polyvinylpyrrolidone
RI: Recognition index
RIPA: Radioimmunoprecipitation assay buffer
RMSD: Root-mean-square deviation
ROS: Reactive oxygen species
RT-PCR: Reverse transcription polymerase chain reaction
SDS: Sodium dodecyl sulfate
SDS-PAGE: Sodium dodecyl sulfate–polyacrylamide gel electrophoresis
SIRT3: Sirtuin 3
SN: Substantia nigra
SOD: Superoxide dismutase
STAT6: Signal transducer and activator of transcription 6
TGF-β: Transforming growth factor-β
TEER: Transepithelial electrical resistance
TEM: Transmission electron microscopy
TH: Tyrosine hydroxylase
TLR: Toll-like receptora
TNF-α: Tumor necrosis factor-α
TP: Total protein
UV: Ultraviolet
WBC: White blood cell count
XRD: X-ray diffraction
YM-1: Chitinase 3-like protein
α-syn: α-synuclein
