# Supplementary material for: Biomimetic nanodelivery system with simultaneous blood–brain barrier-crossing and neuroprotective abilities for anti-parkinsonian therapy
Source: Chin Med. 2025 Nov 8;20:185. doi: 10.1186/s13020-025-01239-2 (PMC12595681; doi:10.1186/s13020-025-01239-2)
Supplement: Supplementary file 1 — Supplementary Material 1. [file 13020_2025_1239_MOESM1_ESM.docx]

**Supporting Information**

**Biomimetic Nanodelivery System with Simultaneous Blood–Brain Barrier-Crossing and Neuroprotective Abilities for Anti-Parkinsonian Therapy**

Xuanying Yin^1, #^, Jinmei Qiu^1, #^, Guowang Cheng^1, #^, Jiaxin Wu^1^, Chen Wang^1^, Chunye Zheng^2^, Shuiqing Huang^1,^ *, Tongkai Chen^1,^ *

*^1^ Science and Technology Innovation Center, Guangzhou University of Chinese Medicine, Guangzhou 510405, China*

*^2^ Department of Neurology, The Second Affiliated Hospital of Guangzhou University of Chinese Medicine, Guangzhou 510120, China*

^#^ These authors contributed equally to this work.

* To whom correspondence should be addressed:

1. Tongkai Chen

Science and Technology Innovation Center, Guangzhou University of Chinese Medicine, 12 Jichang Road, Guangzhou 510405, China

E-mail: chentongkai@gzucm.edu.cn

2. Shuiqing Huang

Science and Technology Innovation Center, Guangzhou University of Chinese Medicine, 12 Jichang Road, Guangzhou 510405, China

E-mail: hsq@gzucm.edu.cn


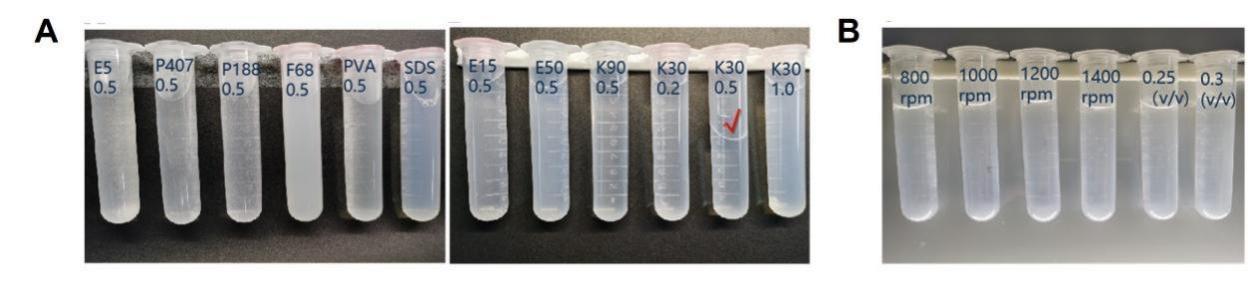


Fig. S1 Preparation of Nar-NCs. (A) The stability of different Nar-NCs formulations when using different stabilizers on Day 1. (B) The stability of different Nar-NCs formulations when using various rotation speeds.


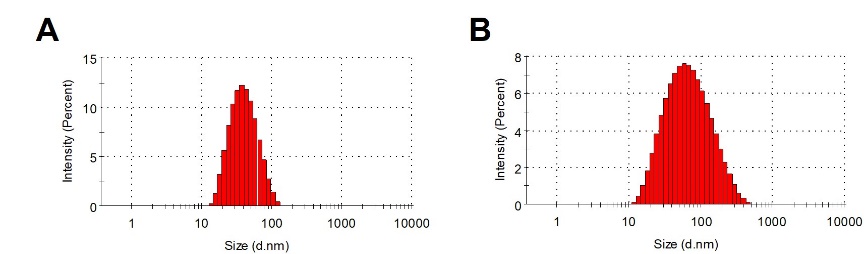


Fig. S2 Particle size distributions of Nar-NCs (A) and DCM@Nar-NCs (B).


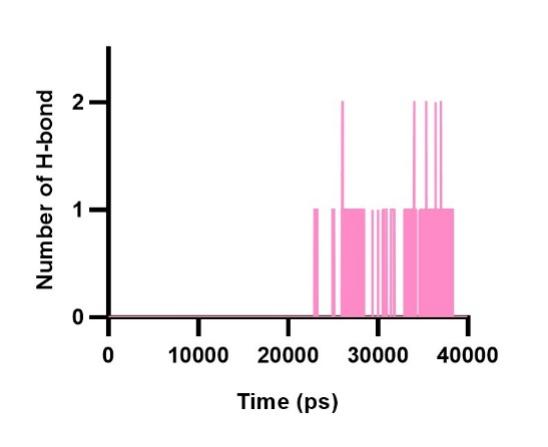


Fig. S3 Numbers of H-bonds between PVP K29/32 and Nar in the conducted molecular dynamics simulation.


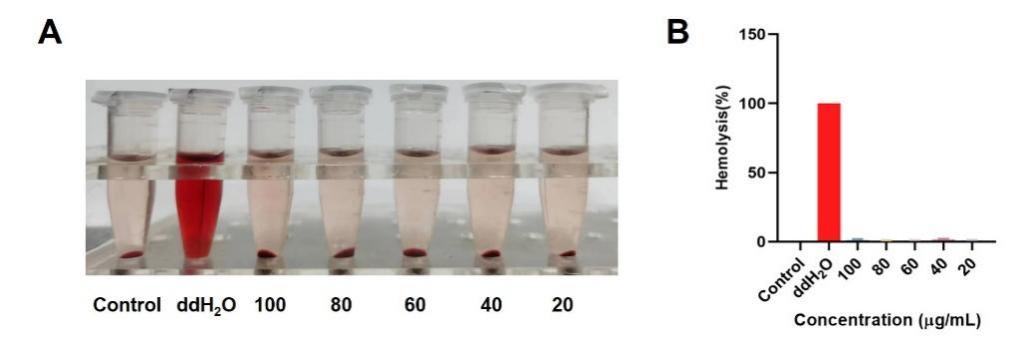


Fig. S4 Study of the hemolytic activity of DCM@Nar-NCs (A) and corresponding quantification (B).


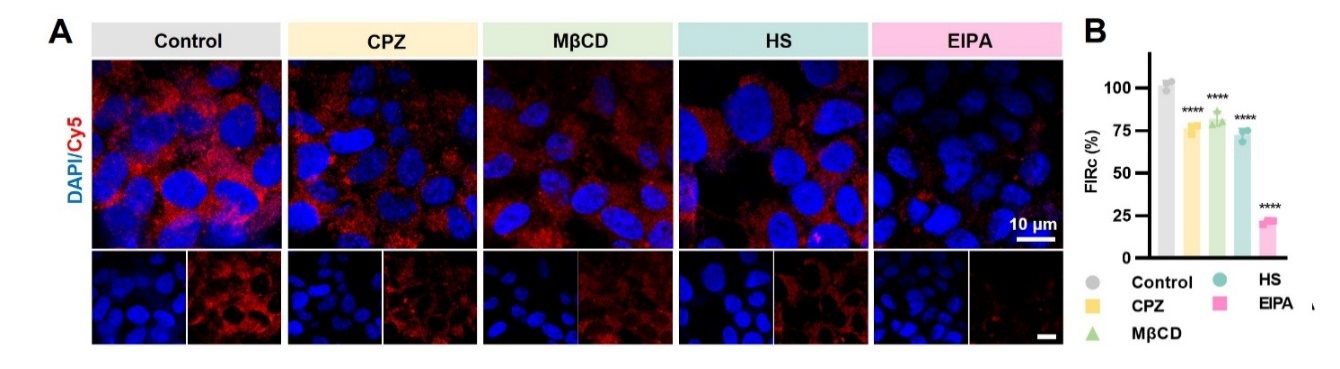


Fig. S5 (A) Representative CLSM images obtained after treatment with different inhibitors in PC12 cells. Scale bar: 10 μm. (B) Fluorescence quantification (n=3). Relative to the Control group: *****P*<0.0001.


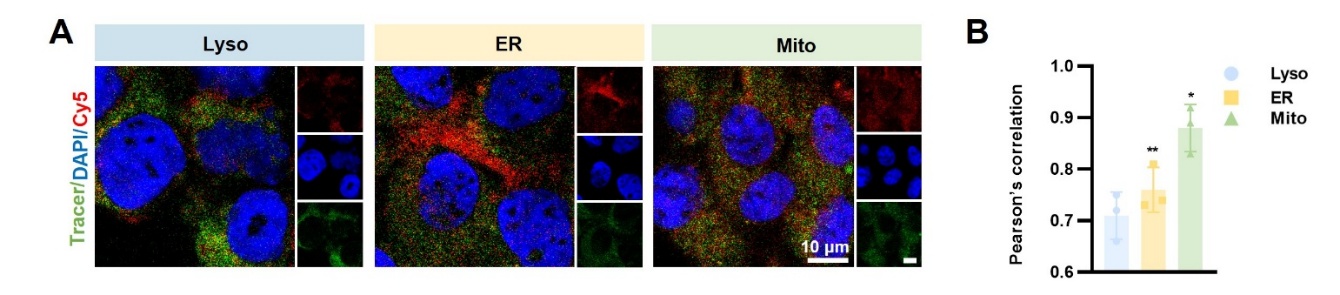


Fig. S6 (A) Colocalization of DCM-Cy5@Nar-NCs with the mitochondria, lysosomes, and endoplasmic reticulum in PC12 cells. Scale bar: 10 μm. (B) Pearson’s correlation of the colocalization (n=3). Relative to the lysosome group: * *P* <0.05, ** *P* <0.01.


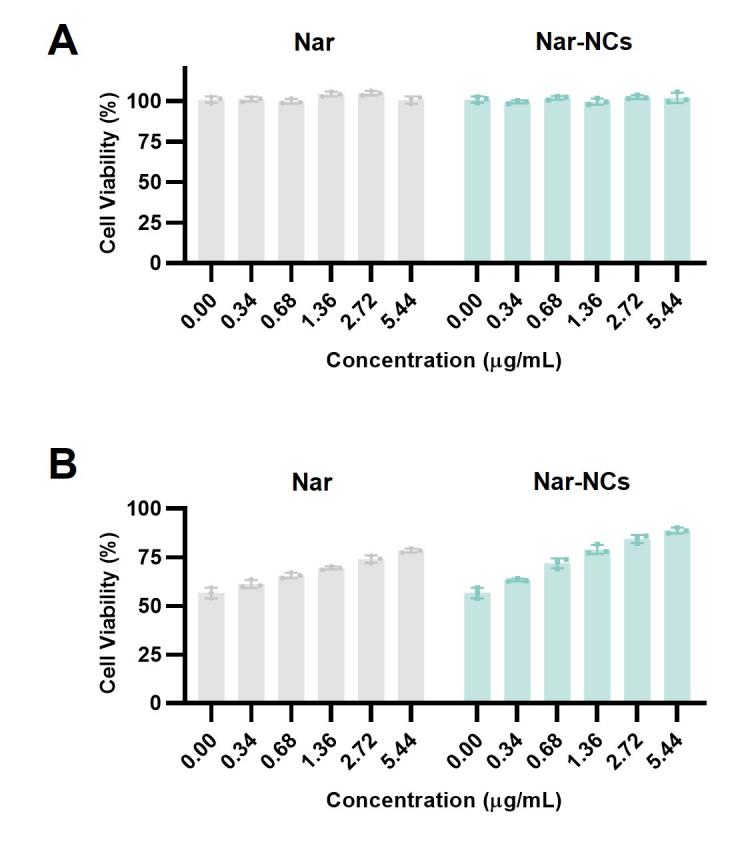


Fig. S7 (A) Evaluation of the cytotoxicity of Nar and Nar-NCs (0.34, 0.68, 1.36, 2.72, and 5.44 μg/mL) when co-incubated with PC12 cells (n=3). (B) Evaluation of the effects of different Nar formulations (0.34, 0.68, 1.36, 2.72, and 5.44 μg/mL) on the viability of MPP^+^-treated PC12 cells (n=3).


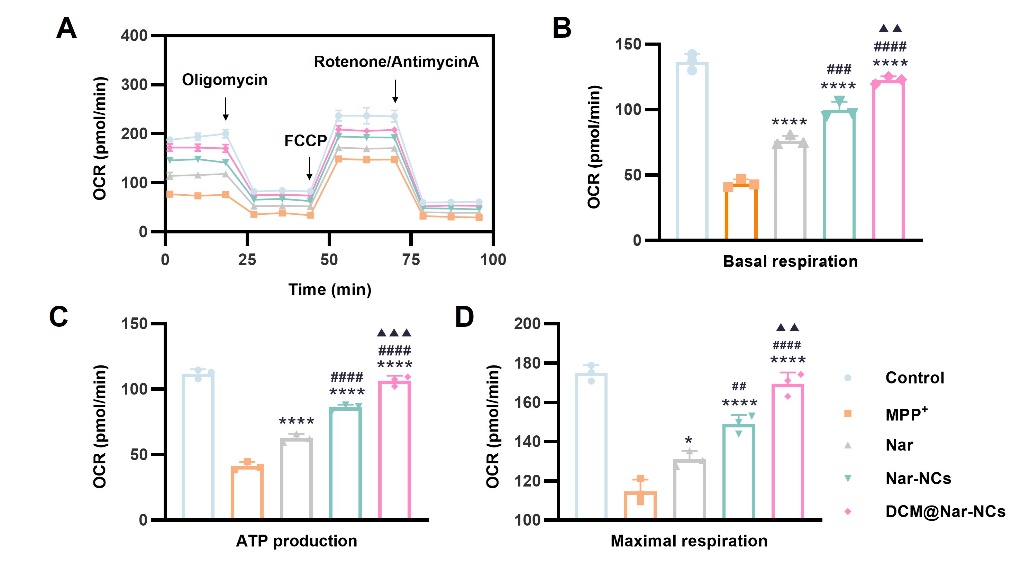


Fig. S8 (A) Oxygen consumption rate (OCR) in PC12 cells subjected to various treatments. (B) Basal respiration (*n*=3). (C) ATP production (*n*=3). (D) Maximal respiration (*n*=3). Versus the MPP^+^ group: **P*<0.05, ***P*<0.01, ****P*<0.001, *****P*<0.0001. Versus the Nar group: ^#^*P*<0.05, ^##^*P*<0.01, ^###^*P*<0.001, ^####^*P*<0.0001. Versus the Nar-NCs group: ^▲^*P*<0.05, ^▲▲^*P*<0.01, ^▲▲▲^*P*<0.001, ^▲▲▲▲^*P*<0.0001.


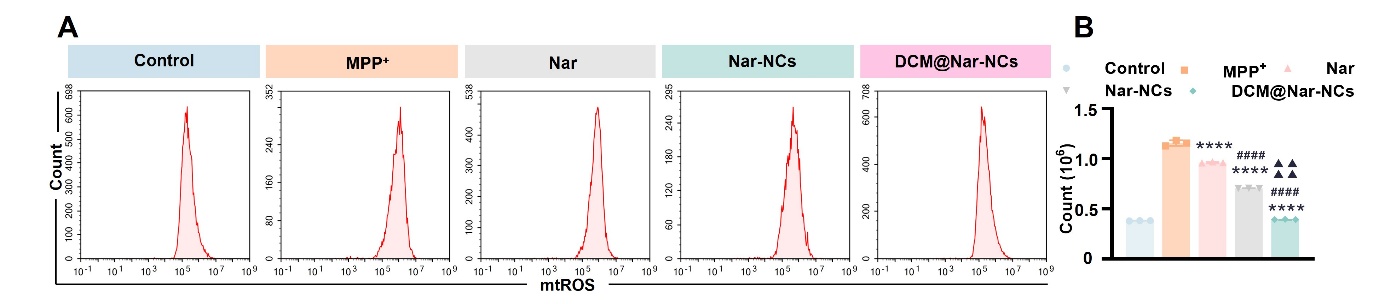


Fig. S9 (A) Mitochondrial ROS (mtROS) generation of different treatments detected by flow cytometry. (B) Corresponding quantitative analysis (*n*=3). Versus the MPP^+^ group: **P*<0.05, ***P*<0.01, ****P*<0.001, *****P*<0.0001. Versus the Nar group: ^#^*P*<0.05, ^##^*P*<0.01, ^###^*P*<0.001, ^####^*P*<0.0001. Versus the Nar-NCs group: ^▲^*P*<0.05, ^▲▲^*P*<0.01, ^▲▲▲^*P*<0.001, ^▲▲▲▲^*P*<0.0001.


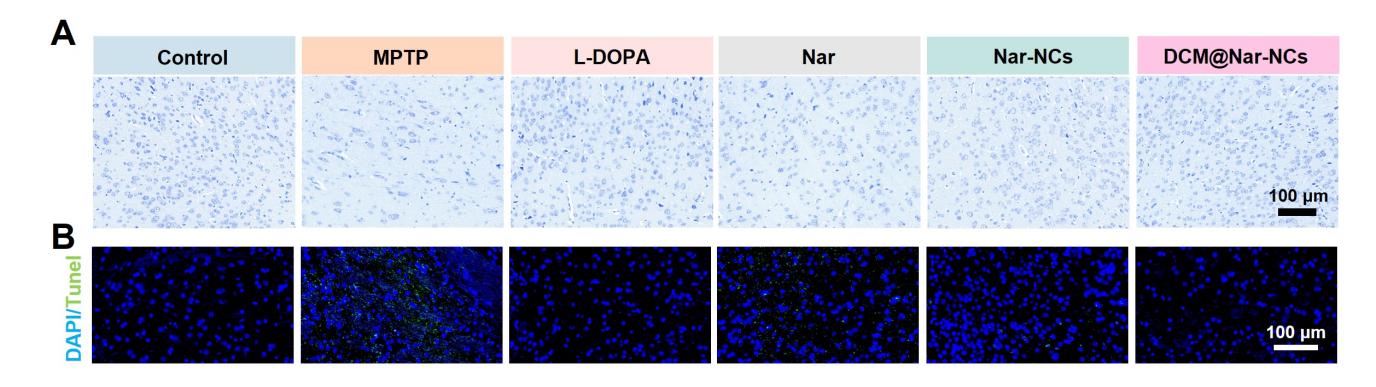


Fig. S10 (A) Nissl staining of cortical neurons from mice in the indicated groups. Scale bar: 100 μm. (B) TUNEL staining of cortical neurons from mice in the indicated groups. Scale bar: 100 μm.


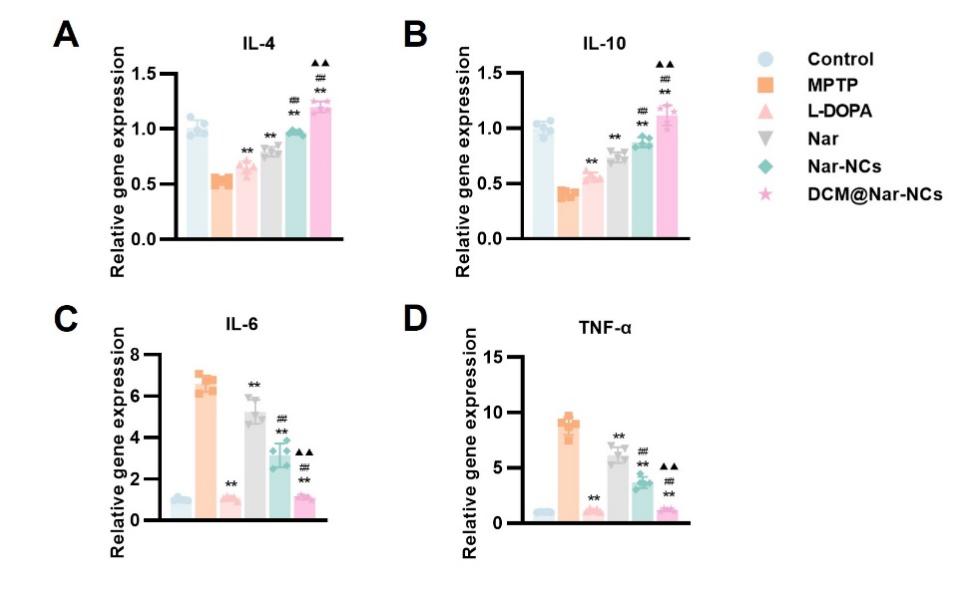


Fig. S11 Quantification of the expression of the anti-inflammatory cytokines IL-4 (A) and IL-10 (B) in the blood of mice from the indicated treatment groups (n=3). Quantification of the expression of the pro-inflammatory cytokines IL-6 (C) and TNF-α (D) in the blood of mice from the indicated treatment groups (n=3). Relative to the MPTP group: **P*<0.05, ***P*<0.01. Relative to the Nar group: ^#^*P*<0.05, ^##^*P*<0.01. Relative to the Nar-NCs group: ^▲^*P*<0.05, ^▲▲^*P*<0.01.


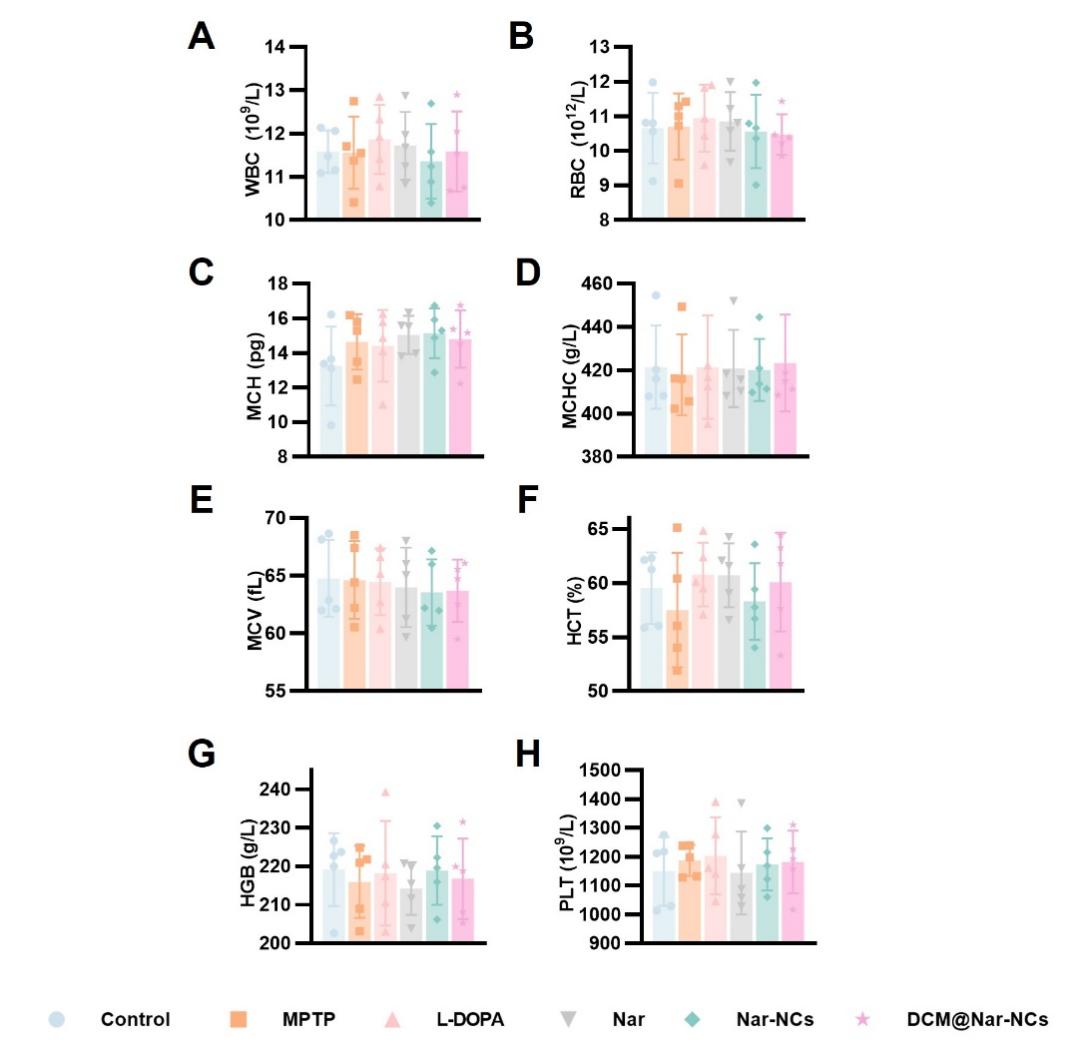


Fig. S12 Routine blood analyses of mice from the indicated treatment groups (n=5). WBC: white blood cells, RBC: red blood cells, MCH: mean corpuscular hemoglobin, MCHC: mean corpuscular hemoglobin concentration, MCV: mean corpuscular volume, HCT: hematocrit, HGB: hemoglobin, PLT: platelets.


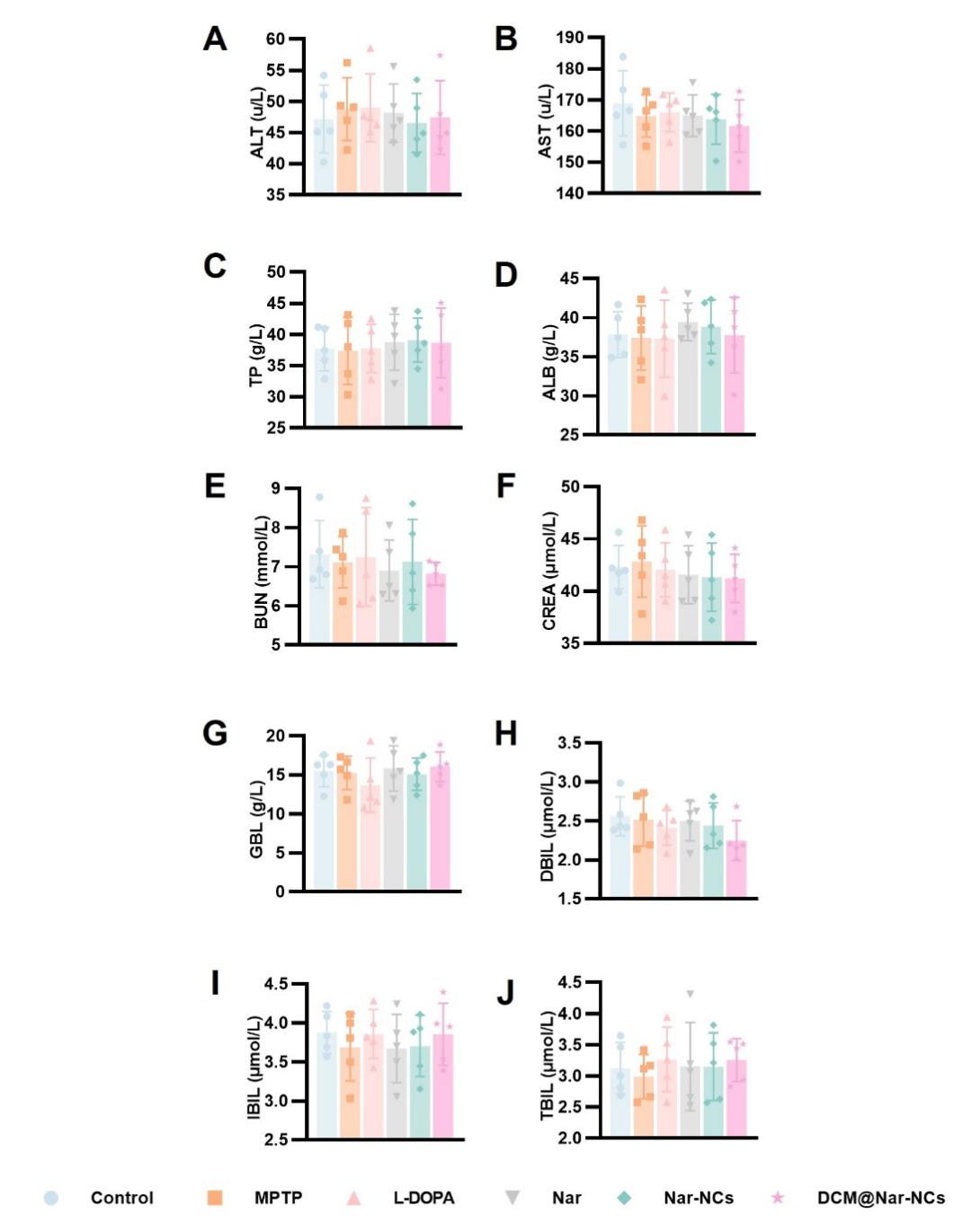


Fig. S13 Biochemical analyses of blood from the indicated treatment groups (n=5). ALT: alanine aminotransferase, AST: aspartate aminotransferase, TP: total protein, ALB: albumin, BUN: blood urea nitrogen, CREA: creatinine, GBL: globulin, DBIL: direct bilirubin, IBIL: indirect bilirubin, TBIL: total bilirubin test.


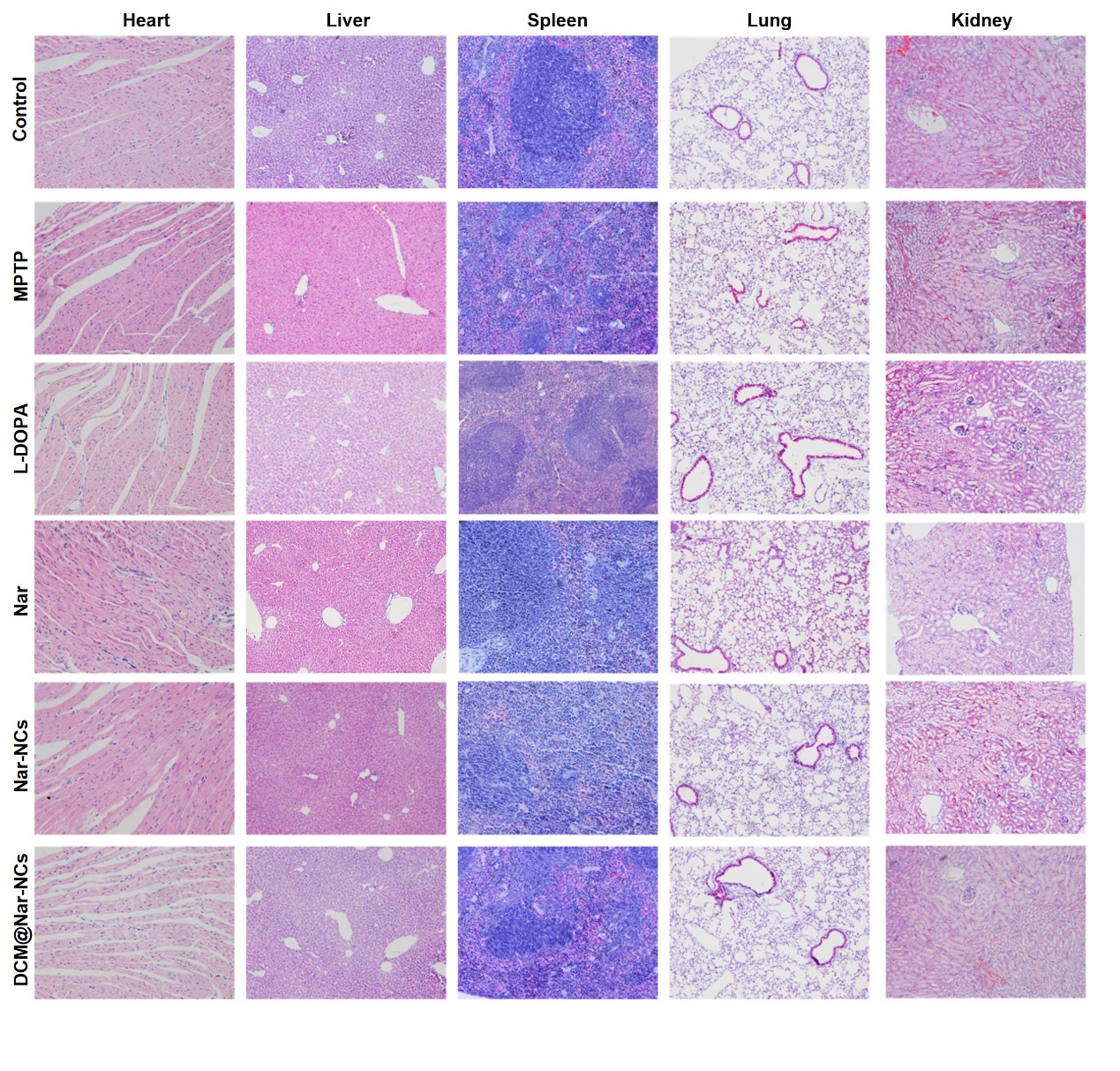


Fig. S14 H&E staining of major organs from mice in the indicated treatment groups.


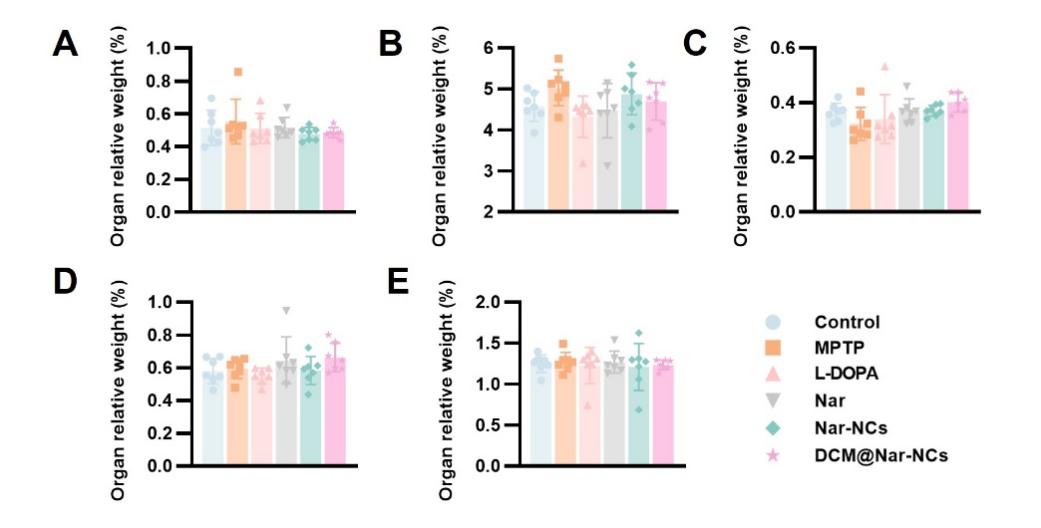


Fig. S15 Relative organ weights for the heart (A), liver (B), spleen (C), lung (D), and kidneys (E) following the indicated treatments (n=7).
